# Using intervention mapping to develop and facilitate implementation of a multifaceted behavioural intervention targeting physical activity and sedentary behaviour in stroke survivors: Physical Activity Routines After Stroke (PARAS): intervention development study

**DOI:** 10.1080/21642850.2022.2066534

**Published:** 2022-05-12

**Authors:** Sarah A. Moore, Darren Flynn, Christopher I. M. Price, Leah Avery

**Affiliations:** a Faculty of Health and Life Sciences, Northumbria University, Newcastle upon Tyne, UK; b Stroke Research Group, Faculty of Medical Sciences, Newcastle University, Newcastle upon Tyne, UK; c Stroke Northumbria, Northumbria Healthcare NHS Foundation Trust, North Shields, Tyne and Wear, UK; d Centre for Rehabilitation, School of Health & Life Sciences, Teesside University, Middlesbrough, UK; e Translational and Clinical Research Institute, Faculty of Medical Sciences, Newcastle University, Newcastle upon Tyne, UK

**Keywords:** Stroke, physical activity, sedentary behaviour, health behaviour change, intervention mapping

## Abstract

**Objectives:**

The benefits of increased physical activity for stroke survivors include improved function and mental health and wellbeing. However, less than 30% achieve recommended physical activity levels, and high levels of sedentary behaviour are reported. We developed a multifaceted behavioural intervention (and accompanying implementation plan) targeting physical activity and sedentary behaviour of stroke survivors.

**Design:**

Intervention Mapping facilitated intervention development. Step 1 involved a systematic review, focus group discussions and a review of care pathways. Step 2 identified social cognitive determinants of behavioural change and behavioural outcomes. Step 3 linked determinants of behavioural outcomes with specific behaviour change techniques (BCTs) to target behaviours of interest. Step 4 involved intervention development informed by steps 1–3. Subsequently, an implementation plan was developed (Step 5) followed by an evaluation plan (Step 6).

**Setting:**

Community and secondary care settings, North East England.

**Participants:**

Stroke survivors and healthcare professionals (HCPs) working in stroke services.

**Results:**

Systematic review findings informed selection of nine ‘promising’ BCTs (e.g. problem-solving). Focus groups with stroke survivors (*n* = 18) and HCPs (*n* = 24) identified the need for an intervention delivered throughout the rehabilitation pathway, tailored to individual needs with training for HCPs delivering the intervention. Intervention delivery was considered feasible within local stroke services. The target behaviours for the intervention were levels of physical activity and sedentary behaviour in adult stroke survivors. Assessment of acceptability and usability with 11 HCPs and 21 stroke survivors/relatives identified issues with self-monitoring tools and the need for a physical activity repository of local services’ and training for HCPs with feedback on intervention delivery. A feasibility study protocol was designed to evaluate the intervention.

**Conclusions:**

A systematic development process using intervention mapping resulted in a multi-faceted evidence- and theory-informed intervention (Physical Activity Routines After Stroke – PARAS) for delivery by community stroke rehabilitation teams.

## Introduction

Low levels of physical activity (Fini et al., 2017) and high levels of time spent sedentary (Morton et al., 2021) are common after stroke. Both are associated with an increased risk of cardiovascular disease and type 2 diabetes, (Bailey et al., 2019) reduced life expectancy (Lee et al., 2012) and impact negatively upon mental health and well-being. Specific stroke-related impairments have been cited as specific barriers to engagement in physical activity and reducing sedentary behaviour, (Nicholson et al., 2012) and may be why exercise preferences appear different in the stroke versus other populations (Banks et al., 2012). This highlights the need for bespoke interventions specifically designed for individuals with stroke.

A wealth of research demonstrates the short-term benefits of structured exercise interventions (commonly delivered in a group format) on cardiovascular risk factors (Moore et al., 2014) and function after stroke; (Saunders et al., 2020) however, longer-term benefits are under-researched. Furthermore, structured exercise interventions for stroke survivors often have little or no emphasis on everyday habitual levels of physical activity and sedentary behaviour (Saunders et al., 2020) and often do not focus on developing stroke survivors’ self-management skills, which prevents them from making sustained changes in behaviour beyond the intervention period (Morris et al., 2015).

The small number of randomised controlled trials (*n* = 9) conducted that target long-term free-living physical activity after stroke show promise (Moore et al., 2018). However, limitations in methodological quality and intervention design prevent any robust conclusions in this field (Moore et al., 2018). Specifically, they lack adequate descriptions of intervention content (Hoffmann et al., 2014) and fidelity assessment, (Bellg et al., 2004) which restricts replicability and prevents successful implementation. There is also a dearth of interventions targeting reductions in sedentary behaviour alongside increasing physical activity of stroke survivors.

Furthermore, few interventions targeting physical activity and sedentary behaviour post-stroke have been developed with reference to theory, systematically developed or robustly evaluated (Moore et al., 2018). The application of health behaviour change theory is critically important to gain a thorough understanding of the antecedents of the behaviours of interest, to develop targeted and effective interventions (Nicholson et al., 2012, 2014). Theory based interventions have been shown to significantly impact upon physical activity behaviour (Gourlan et al., 2016). Intervention Mapping is a practical framework for systematic, evidence and theory-based planning for behavioural change (Kok, 2014) and has been particularly effective in the context of healthcare, (Fernandez et al., 2019; Hurley et al., 2016) including informing the development of interventions targeting physical activity behaviour (Brug, Oenema, & Ferreira, 2005).

Using Intervention Mapping, our aim was to systematically develop an evidence-and theory-informed behavioural intervention targeting long-term, free-living physical activity and sedentary behaviour for integration into the stroke rehabilitation care pathway.

## Methods

### Overview of development process

**Figure 1.**
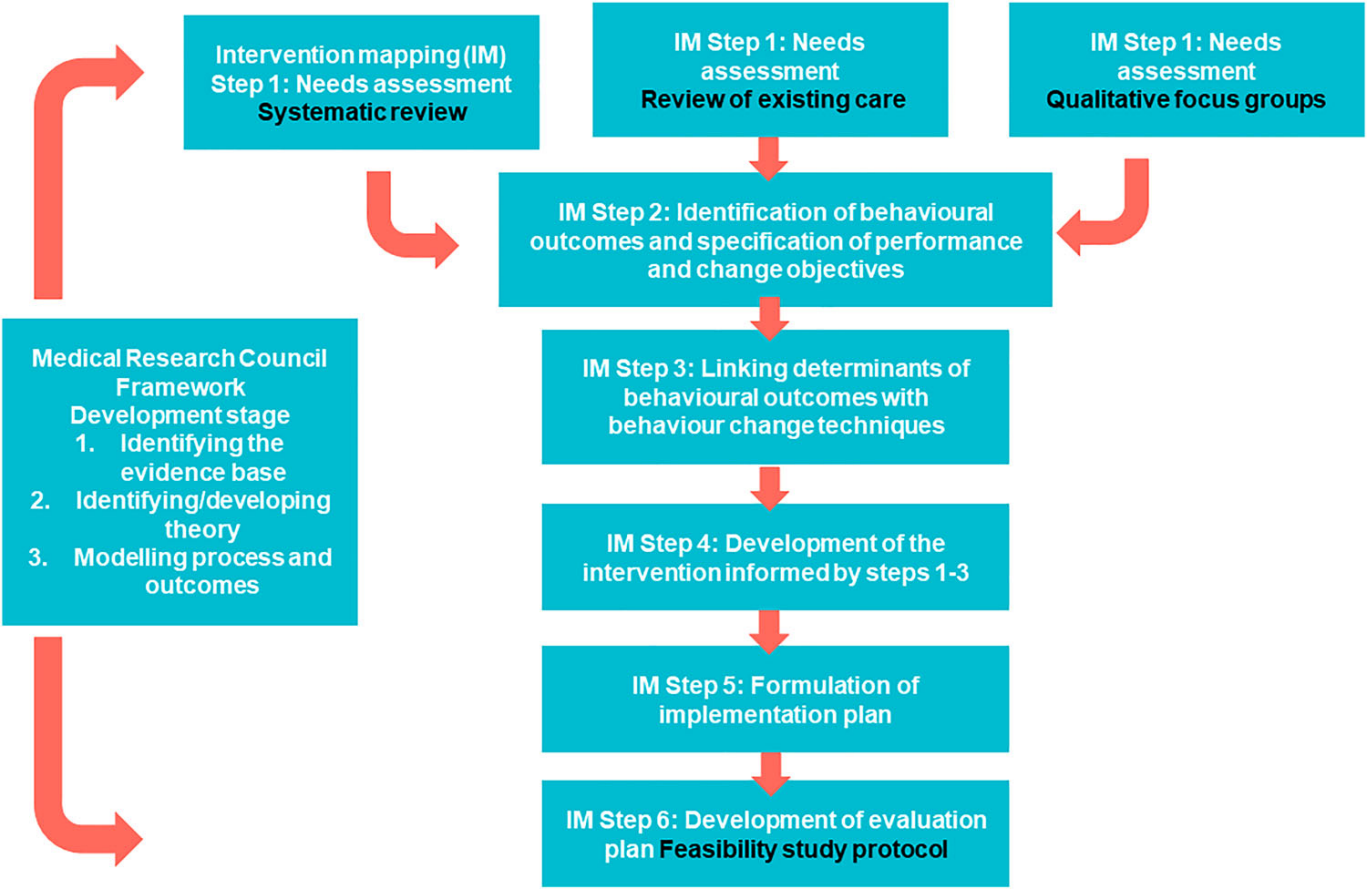
PARAS development process.

Our intervention was developed with reference to the Medical Research Council (MRC) Framework for the Development and Evaluation of Complex Interventions. Complex interventions are those that contain several interacting components. The current intervention was defined as complex given the complex needs of the target population (i.e. stroke impairment) and the challenges associated with and influencing behavioural change. These complexities present unique problems for evaluation (Craig et al., 2013). Alongside the MRC framework, we utilised Intervention Mapping to inform intervention content, delivery, implementation and evaluation (Bartholomew, Parcel, Kok, Gottlieb, & Fernandez, 2011). Details of how the six steps of the implementation mapping intervention development approach described by (Bartholomew et al., 2011) were applied are described in the methods and summarised in Figure 1.

## Methods and results

## Ethics statement

Ethical approval for the development workshops with stroke survivors and healthcare professionals was givenon the 24/10/2016 from the Faculty of Medical Science ethics committee at Newcastle University, Newcastle upon Tyne, U.K. (reference number 01211/2016). Fully informed written consent was gained from all participants who took part in the study.

### Step 1: needs assessment

To enable the development and implementation of a tailored intervention to effectively target physical activity and sedentary behaviour after stroke, we conducted a needs assessment. To do this, we first conducted a systematic review of the literature (Moore et al., 2018) and secondly consulted with healthcare professionals (HCPs) and stroke survivors and identified local community stroke care pathways (where they existed) to determine how a new intervention could integrate with and/or complement current pathways.

### Systematic review

A previously published systematic review of randomised controlled trials was undertaken to explore the characteristics and promise of existing intervention components targeting free-living physical activity and/or sedentary behaviour after stroke (Moore et al., 2018).

### Key findings of systematic review

All nine studies (*N* = 719 participants) included in the systematic review targeted physical activity behaviour and none targeted sedentary behaviour. Six of the interventions evaluated were rated as promising – i.e. interventions with statistically significant between- or within-group improvements in outcomes greater than those of the control or comparator group (Gardner et al., 2016). All of these interventions involved an element of supervised support that was tailored to individual needs. Both face-to-face and telephone contact were identified as promising modes of intervention delivery. The number of contacts for promising interventions ranged from a single contact to 36 contacts, with the duration of interventions ranging from one single contact to twelve consecutive weeks. Nine promising behaviour change techniques (BCTs) were identified with reference to the BCT Taxonomy V1 (Michie et al., 2013) and considered for inclusion in our intervention: information about health consequences; information about social and environmental consequences; goal-setting behaviour; problem-solving; action planning; feedback on behaviour; biofeedback; social support unspecified; and credible source.

Although the systematic review identified some promising interventions and associated components, it highlighted the need for the development of a novel intervention that addressed previous methodological and theoretical limitations and that incorporated stroke survivor and HCP preferences.

### Qualitative focus group discussions

A series of interactive focus group discussions were conducted with stroke survivors and HCPs. Stroke survivors were recruited by advert within the North East of England. Eligibility criteria were broad and included stroke diagnosis, community dwelling and able to access and attend focus group discussions. HCPs from five NHS North East England stroke rehabilitation services were invited to take part in a focus group discussion. Eligibility criteria for HCPs were: Qualified physiotherapist, technical instructor, physiotherapy assistant; working in the NHS; currently working in stroke rehabilitation; and able to access and attend the focus group. Participants were recruited until it was felt that data saturation had been reached. The overall aim of the focus groups was to identify determinants of behavioural change for both stroke survivors and HCPs and to explore the barriers and enablers to engage in long-term physical activity and reduction of sedentary behaviour. A secondary aim was to add specific context to the findings of our systematic review, and to explore preferences around the intervention components identified by the systematic review. Focus group discussions were conducted in person, audio recorded and transcribed verbatim. Topic guides were developed using the COM-B model exploring how capability, opportunity and motivation interact to influence behaviour. A presentation explaining the overall aim of the focus group, definitions of physical activity and sedentary behaviour and the benefits of post-stroke activity was delivered at the start of each focus group. Open-ended questions about pre- and post-stroke activity levels, and barriers and enablers to engaging in physical activity and reducing sedentary behaviour after stroke were subsequently explored with stroke survivors. HCPs were asked about motivators, barriers and facilitators to supporting stroke survivors to be more physically active and reduce sedentary behaviour. Each focus group lasted approximately one and a half hours.

Data were analysed using the Theoretical Domains Framework (TDF) (Cane, O’Connor, & Michie, 2012) to facilitate an exploration of behavioural determinants likely to predict and impact upon behaviour and behavioural change. Three researchers (SAM, a clinical academic stroke physiotherapist, LA, a chartered health psychologist with expertise in health behaviour change and qualitative research methods and a master’s degree student) read, re-read and analysed transcripts following the conduct of each focus group discussion. Any unsubstantiated issues or points were explored further during subsequent group discussions (i.e. topic guides were revised accordingly). The skill mix of the researchers ensured appropriate questions were asked and responses were further probed for comprehensive understanding. Analyses of the data involved assigning text segments to one or more domains of the TDF and generating themes within each domain. All focus group transcripts with HCPs and stroke survivors were coded and analysed by hand, i.e. no qualitative software was used. Common themes across the stroke survivors and HCPs were subsequently established with regular meetings held between researchers to discuss independent analyses and gain consensus.

### Findings of focus groups discussions

Eighteen stroke survivors (11 male, median age 64 years, interquartile range (IQR) 15, time since stroke 52 months, IQR 39, 15 ambulatory (with or without walking aid), 3 wheelchair users) and 24 HCPs (physiotherapists *n* = 14, technical instructors *n* = 8, physiotherapy assistants *n* = 2, working on the ward *n* = 5, working across ward and community *n *= 3, working in the community/outpatients *n* = 16) participated across 7 focus groups. All 14 of the theoretical domains of the TDF were identified from the data generated from stroke survivor focus groups. The most saliant domains were ‘environmental context and resources’, ‘beliefs about consequences’ and ‘beliefs about capabilities’ (Stroke survivor TDF domains themes with related change objectives are presented in Table 1, Step 2).

**Table 1.** Stroke survivor behavioural outcomes, performance objectives and change objectives related to theoretical domains and associated themes identified from focus group data.

**Stroke survivor behavioural outcomes***To develop knowledge to raise awareness of the importance of physical activity and reducing sedentary behaviour in the context of stroke, and skills to increase and sustain activity levels and reduce sedentary behaviour to enable performance of activities of daily living*. **Performance objectives** Understands benefits of physical activity and reducing sedentary behaviour after stroke Requests support to increase physical activity and reduce sedentary behaviour at the most appropriate time Selects and safely performs meaningful and sustainable physical activity and/or reduces sedentary behaviour Identifies and utilises social support to maintain physical activity behaviour and reduce sedentary time Applies behavioural goal setting, action planning and coping planning to selected physical activities and/or reducing sedentary behaviour Selects methods of self-monitoring physical activity and sedentary behaviour Self-monitors physical activity and sedentary behaviour, behavioural goal attainment and associated confidence and well-being Plans methods for maintaining physical activity or reducing sedentary behaviour		
**TDF Domain**	**Themes identified**	**Stroke survivor change objectives**
**Knowledge**	Timing of information provision is important but highly individual Amount and intensity of physical activity has to increase to derive benefit Lack of knowledge about physical activity opportunities and support available prevents engagement	To have the knowledge and confidence to request information at the right time To develop skills and mastery of tools and resources to safely self-manage physical activity and sedentary behaviour
**Skills**	Planning and self-monitoring can facilitate engagement in physical activity and help reduce sedentary time	To have capacity and capability to master self-monitoring of physical activity
**Social/Professional role**	Past physical activity levels and engagement facilitates participation in physical activity and physical activity choices Being physically active with people of a similar age and varying abilities provides confidence	To identify meaningful physical activities To identify and engage social support
**Beliefs about capabilities**	Confidence about ability is a barrier to increasing physical activity Old age, comorbidities and fatigue limit ability to be active	To develop knowledge and capability to confidently undertake physical activity. This involves articulating reasons for change. To be able to describe personal barriers to physical activity and reducing sedentary behaviour and identify potential solutions.
**Optimism**	A positive attitude facilitates participation in physical activity and reduces sedentary behaviour when prompted	To be able to set behavioural goals and actions and monitor well-being when goals are achieved
**Beliefs about consequences**	Too much physical activity too soon could lead to further health problems including stroke recurrence Physical activity as a mechanism to return to pre-stroke self Fear of falls is a barrier Physical activity gets you out of the house Physical activity fills time post stroke	To be able to identify a physical activity that feels safe but is effective for achieving outcome goals To be able to identify meaningful, safe physical activity outcomes and select appropriate activities likely to lead to these outcomes
**Reinforcement**	Sense of achievement can facilitate longer-term physical activity	To identify and use appropriate tools to measure physical activity against behavioural and outcome goals To monitor wellbeing in response to goal attainment
**Intentions**	Recognition of the importance of physical activity but HCP information provision not sufficient to enable physical activity	To access appropriate physical activity information and advice and select meaningful activities that are more likely to lead to behavioural change
**Goals**	Planning physical activity in advance increases the likelihood it will be undertaken	To set appropriate and realistic behavioural and outcome goals
**Memory, attention and decision processes**	Having to think about everything before doing it post-stroke makes engagement in physical activity more difficult	To use appropriate tools to set realistic goals and action plan to aid memory
**Environmental context and resources**	Stroke specific groups provide emotional and physical support that can facilitate physical activity. Modifying home environment can facilitate physical activity and reduce sedentary time Restricted car use can impact on physical activity levels Mixed ability and co-morbidity groups can facilitate physical activity Knowledge and skills of professionals can affect uptake of physical activity Lack of longer-term physiotherapy input can affect long-term physical activity Lack of information provision, resources and available options is a barrier to physical activity Dependence on others to be able to leave home is a barrier to increasing physical activity	To identify and engage social support to enable PA To understand and access resources available within the home and locally to increase physical activity and reduce sedentary time
**Social influences**	Peer support groups provide a means of support and can help to facilitate physical activity	To identify and engage with social support
**Emotion**	Group based activities that are enjoyable and provide a means of support can facilitate physical activity Anxiety and depression is a barrier to engaging on activities	To select safe physical activities that lead to meaningful outcomes and a sense of well-being
**Behavioural regulation**	Planning and self-monitoring can facilitate engagement in physical activity and help reduce sedentary time	To use planning and self-monitoring tools meaningfully and review physical activity and sedentary behaviour to support maintenance

Data generated from HCP focus groups identified seven theoretical domains: ‘knowledge’, ‘skills’, ‘social/professional role and identity’, ‘belief about consequences’, ‘beliefs about capabilities’, ‘reinforcement’ and ‘environmental context and resources’. The most saliant domains that emerged from the data were ‘environmental context and resources’ and ‘skills’ (HCP TDF domains themes with related change objectives are presented in Table 2, Step 2).

**Table 2.** Healthcare professional behavioural outcomes, performance objectives and change objectives alongside theoretical domains and associated themes identified from focus group data.

***HCP behavioural outcomes:****To improve/increase knowledge about the benefits of physical activity and reducing sedentary behaviour in the context of stroke and to develop skills to promote and sustain physical activity levels and reduce sedentary behaviour to enable stroke survivors to perform activities of daily living.*		
**Performance objectives**: Accepts supporting physical activity and reducing sedentary behaviour after stroke is beneficial for stroke rehabilitation and part of the HCP role Supports stroke survivors to successfully engage in the PARAS intervention Appropriately uses PARAS intervention resources to support stroke survivors to engage with the PARAS intervention Appropriately uses behaviour change counselling techniques to support stroke survivor’s to identify reasons for physical activity behaviour change and maintenance		
**Theoretical Domains**	**Themes**	**HCP change objectives**
**Knowledge**	Training in the benefits and use of physical activity in the context of stroke rehabilitation would be beneficial	To understand the benefits of physical activity and reducing sedentary behaviour post-stroke To deliver person centred, personalised support to stroke survivors
**Skills**	Being able to promote physical activity tailored to individual needs is essential for promoting participation Skills to help patients overcome barriers to physical activity will facilitate longer-term engagement	Able to identify physical activity resources available to stroke survivors that meet their individual needs To support stroke survivors to undertake barrier identification and coping planning
**Social/Professional role**	Promoting physical activity is part of the healthcare professional’s role	Engage in training to use a range of behavioural tools to target physical activity
**Beliefs about capabilities**	Engaging patients in physical activity and reducing sedentary time is difficult when pre-stroke activity levels were low	To apply appropriate behaviour change counselling techniques to engage stroke survivor in behaviour change and maintenance
**Beliefs about consequences**	Being overweight can be a barrier to physical activity and reduction in sedentary time for stroke survivors Motivational strategies will only work on a proportion of patients Withdrawal of physiotherapy support makes promotion and support of long-term physical activity problematic	Able to identify the different barriers to behaviour change and support the formulation of appropriate coping plans Able to succeed with resistant or ambivalent stroke survivors To understand how to support stroke survivor’s long-term physical activity within the context of individual service delivery, identifying meaningful, sustainable activities and social support
**Reinforcement**	Seeing patients succeed is an incentive to promoting physical activity	Able to support stroke survivors to identify and achieve meaningful, sustainable physical activities and social support
**Environmental context and resources**	Increased availability of physical activity options for patients post stroke would be beneficial Community physical activity groups are usually targeted at higher functioning stroke survivors Promoting long-term engagement in physical activity can be difficult without an individual in the team to take on this role Tools to tailor physical activity to individual needs would be beneficial Self-monitoring tools and technologies could be useful to initiate and maintain physical activity. Funding and time are barriers to physiotherapists participating in training to effectively target and support stroke survivors to be physically active	To develop knowledge of physical activity resources available to stroke survivors To be able to adapt physical activity support to individual needs of stroke survivors and enable identification of meaningful sustainable activities and goals To be able to access and effectively apply tools to support engagement in physical activity and reduction in sedentary behaviour e.g. self-monitoring tools To be able to access training on how to support physical activity and reduce sedentary behaviour post-stroke within restrictions of current job role

Although the aim of the focus group discussions with stroke survivors was to explore the determinants of behavioural change in relation to physical activity *and* sedentary behaviour, participants focused their discussions on physical activity highlighting a potential lack of understanding and awareness about sedentary behaviour and the role it plays in stroke rehabilitation. As such, a need for the development of an intervention to target sedentary behaviour as well as physical activity behaviour was identified.

Lack of sustainable physical activity options was identified as a key barrier by stroke survivors with a lack of timely information and long-term support also reported. Enablers to increasing physical activity and reducing sedentary behaviour were identifying meaningful, accessible, sustainable activities with social support and developing skills for self-monitoring physical activity and well-being, e.g. linking a change to behaviours with improvements in activities of daily living were considered vitally important. HCPs also identified environmental context and access to resources as barriers to promoting physical activity and reducing sedentary behaviour, as well as a lack of skills to effectively support behaviour change, particularly when stroke survivors presented significant barriers and challenges.

### Exploration of intervention opportunities within existing pathways

To identify current stroke rehabilitation services and explore potential for delivering the intervention within existing pathways, a questionnaire was sent to North East England community stroke teams. Questions explored current and future staffing, current service provision, and potential for participation in a future intervention study. Community stroke services at seven North East NHS Trusts were considered for inclusion. Four of these Trusts were already involved in another rehabilitation study led by the research team; therefore it was agreed that they would not be approached to reduce burden.

### Key findings of exploration of existing pathways

Participating trusts did not report any specific interventions or resources already in place to target free-living physical activity and sedentary behaviour and reported having an interest, capacity and manager agreement to take part in a future feasibility study (Appendix A).

### Key findings of needs assessment

The needs assessment highlighted that physical activity and sedentary behaviour are not adequately addressed post-stroke and HCPs do not feel equipped to target these behaviours effectively. Findings indicated that the intervention should be adaptable to individual needs, circumstances and preferences. A supported self-management approach was considered most appropriate to target these requirements. Mapping of existing stroke rehabilitation pathways revealed that there was a potential to incorporate a physical activity and sedentary behaviour intervention and training for HCP into current practice.

### Step 2: identification of behavioural outcomes, and specification of performance and change objectives

The needs assessment conducted in Step 1 identified the behaviours to target with our intervention: Stroke survivors – physical activity and sedentary behaviour, HCPs – consultation behaviour including knowledge about physical activity and sedentary behaviour in the context of stroke and skills to effectively target behaviour change. The two behavioural outcomes of the PARAS intervention, related performance objectives (tasks required) and change objectives (i.e. aspects of behaviour individuals are required to learn, do or change) that need to be accomplished by stroke survivors and HCPs in order to achieve the behavioural outcomes and performance objectives were developed with reference to the TDF domains and domain-specific themes identified in Step 1. These are described in Tables 1 and 2.

### Step 3: selection of theory-based intervention content

Selection of the theories/models to underpin the behaviour change intervention were informed by the findings of steps 1 and 2.

### Theoretical underpinning of the stroke survivor component of the intervention

Two theories were selected to underpin the stroke survivor component of the multifaceted intervention, the Health Belief Model (Rosenstock, Strecher, & Becker, 1998) and Self-Regulation Theory (Leventhal, Brissette, & Leventhal, 1997). The Health Belief Model assumes an individual’s belief in the personal threat of an illness together with their belief that the effectiveness of a health behaviour or action will determine whether they change their behaviour (or not). Self-regulation Theory involves guiding an individual’s own thoughts, behaviours and feelings to reach the goals. It was felt that these two models/theories in combination were appropriate with specific reference to the findings generated by the qualitative research.

Step 1 informed theory selection to target individual perceptions of stroke and stroke recurrence including the use and perceived benefits and disadvantages of physical activity and inactivity. It was felt that the selected model was appropriate particularly around challenging beliefs about the consequences of physical activity/inactivity and as such formulate reasons/intentions for engaging in physical activity.

Self-Regulation Theory assumes that behaviour is goal-directed or purposive. Findings from our systematic review and focus group discussions supported the need for specific strategies to target volition as well as motivation in recognition that maintenance of physical activity for stroke survivors can be particularly challenging given the level of cognitive and physical effort required. Furthermore, inclusion of several specific BCTs that target self-regulation e.g. goal-setting behaviour; problem-solving; action planning; feedback on behaviour were identified from the systematic review as promising.

Behaviour change techniques are the irreducible active ingredients of interventions targeting behaviour change, and are useful to inform, describe, deliver and evaluate behaviour change interventions as well as operationalising underpinning theory (Michie et al., 2015). TDF domains were identified from the data generated from stroke survivor focus group discussions and BCTs were selected with reference to those domains, supported by evidence from the systematic review (i.e. BCTs identified by the review as promising). When discrepancies occurred between the findings of the qualitative study and the systematic review, research team discussion led to a consensus agreement in terms of inclusion/exclusion of specific BCTs. The outcome of the decision-making process is summarised in Table 3 which also describes theoretical constructs targeted and intervention components which were further developed in Step 4.

**Table 3.** Theoretical intervention mapping targeting physical activity and sedentary behaviour of stroke survivors.

TDF Domain	Behaviour change technique (used/not used, promising/non-promising from systematic review findings)	Selection rationale	Theoretical constructs targeted and potential intervention components
**Knowledge**	5.1: Information about health consequences (used and promising) **Definition:** Provide information (e.g. written, verbal, visual) about health consequences of performing the behaviour	Assessed as promising systematic review Supported by qualitative findings Expert consensus	Appropriate theory: Health Belief Model (HBM)Constructs: All constructs of HBMSuggested/example intervention component(s): Booklet for patients and/or DVD containing information and patient narratives Access to repository of information via HCPs to obtain details of local activities, support and resources
	5.2: Salience of consequences (not used) **Definition:** Use methods specifically designed to emphasise the consequences of performing the behaviour with the aim of making them more memorable (goes beyond informing about consequences)	Not supported by systematic review findings, but overruled based on small sample sizes Supported by qualitative findings Expert consensus	
	3.1: Social support (unspecified) (used and promising) **Definition:** Advise on, arrange or provide social support *(e.g. from friends, relatives, colleagues’, buddies’ or staff)* or non-contingent praise or reward for performance of the behaviour*.* It includes encouragement and counselling, but only when it is directed at the behaviour	Supported by systematic review findings Supported by qualitative findings Expert consensus	
	9.1 Credible source (used promising) **Definition:** Present verbal or visual communication from a credible source in favour of or against the behaviour	Supported by systematic review findings Supported by qualitative findings Expert consensus	
**Skills**	4.1: Instruction on how to perform the behaviour (used and non-promising) **Definition:** Advise or agree on how to perform the behaviour (includes ‘Skills training’)	Not supported by systematic review findings, overruled based on small sample sizes and the need for instruction on how to perform specific activities safely Supported by qualitative findings Expert consensus	Relevant theory: Self-Regulation Theory (SRT)Constructs: Action planning, problem solvingSuggested/example intervention component(s): Workbook (template to be completed/populated in discussion with a HCP) and/or DVD
	6.1: Demonstration of the behaviour (used and non-promising) **Definition:** Provide an observable sample of the performance of the behaviour, directly in person or indirectly	Not supported by systematic review findings, overruled based on the need to demonstrate behaviour for safety Supported by qualitative findings Expert consensus	
**Social/Professional role**	3.1: Social support (unspecified**)** (used and promising) **Definition:** Advise on, arrange or provide social support *(e.g. from friends, relatives, colleagues’, buddies’ or staff)* or non-contingent praise or reward for performance of the behaviour*.* It includes encouragement and counselling, but only when it is directed at the behaviour	Supported by systematic review findings Supported by qualitative findings Expert consensus	Relevant theory: SRT Constructs: Feedback; problem solving Suggested/example intervention component(s): Access to repository of information via HCPs to obtain details of local support and resources. Constructs are targeted by social support (e.g. positive reinforcement and sharing of information to overcome barriers).
**Beliefs about capabilities**	1.1: Goal setting (behaviour) (used and promising) **Definition:** Set or agree on a goal defined in terms of the behaviour to be achieved	Supported by systematic review findings Supported by qualitative findings Expert consensus	Relevant theory: HBM & SRT Constructs: Individual perceptions; likelihood of action; goal setting; problem solving Suggested/example intervention component(s): Information booklet and/or DVD concentrating on antecedents and pros and cons for changing behaviour. Booklet template to be completed in discussion with a HCP targeting goal setting and problem solving.
	1.2: Problem solving (used and promising) **Definition:** Analyse, or prompt the person to analyse, factors influencing the behaviour and generate or select strategies that include overcoming barriers and/or increasing facilitators (includes ‘Relapse Prevention*’ and ‘*Coping Planning*’*)	Supported by systematic review findings Supported by qualitative findings Expert consensus	
	4.2: Information about antecedents (not used) **Definition:** Provide information about antecedents (*e.g. social and environmental situations and events, emotions, cognitions)* that reliably predict performance of the behaviour e.g. Advise to keep a record of snacking and of situations or events occurring prior to snacking	Not used in systematic review, overruled based on strength of qualitative findings Supported by qualitative findings Expert consensus	
**Optimism**	5.4: Monitoring of emotional consequences (not used) **Definition:** Prompt assessment of feelings after attempts at performing the behaviour	Not used in systematic review, overruled based on strength of qualitative findings Supported by qualitative findings Expert consensus	Relevant theory: HBM & SRTConstructs: Modifying factors; self-monitoringSuggested/example intervention component(s): Booklet to be completed/populated in discussion with a HCP, reviewed and feedback provided to provide positive reinforcement
**Beliefs about consequences**	5.1: Information about health consequences (used and promising) **Definition:** Provide information (e.g. written, verbal, visual) about health consequences of performing the behaviour	Supported by systematic review findings Supported by qualitative findings Expert consensus	Relevant theory: HBM Constructs: Individual factors; modifying factors Suggested/example intervention component(s): Information booklet and/or DVD with patient and HCP narratives
	5.3: Information about social and environmental consequences **(**used and promising) **Definition:** Provide information (e.g. written, verbal, visual) about social and environmental consequences of performing the behaviour	Supported by systematic review findings Supported by qualitative findings Expert consensus	
**Reinforcement**	10.4: Social reward (not used) **Definition:** Arrange verbal or non-verbal reward if and only if there *has been* effort and/or progress in performing the behaviour (includes ‘Positive reinforcement’)	Not used in systematic review, overruled based on strength of qualitative findings Supported by qualitative findings Expert consensus	Relevant theory: SRTConstructs: FeedbackSuggested/example intervention component(s): Feedback from a HCP or social group relating to attainment of goals
**Intentions**	5.1: Information about health consequences (used and promising) **Definition:** Provide information (e.g. written, verbal, visual) about health consequences of performing the behaviour 5.3: Information about social and environmental consequences **(**used and promising) **Definition:** Provide information (e.g. written, verbal, visual) about social and environmental consequences of performing the behaviour	Supported by systematic review findings Supported by qualitative findings Expert consensus	Relevant theory: HBMConstructs: Individual factors; modifying factorsSuggested/example intervention component(s): Information booklet and/or DVD with patient and HCP narratives
**Goals**	1.1: Goal setting (behaviour) (used and promising) **Definition:** Set or agree on a goal defined in terms of the behaviour to be achieved	Supported by systematic review findings Supported by qualitative findings Expert consensus	Relevant theory: SRT Constructs: Goal setting Suggested/example intervention component(s): Goal setting component within the booklet. Template to be completed in discussion with a HCP
**Memory, attention and decision processes**	8.3: Habit formation (not used) **Definition:** Prompt rehearsal and repetition of the behaviour in the same context repeatedly so that the context elicits the behaviour	Not used in systematic review, overruled based on strength of qualitative findings Supported by qualitative findings Expert consensus	Relevant theory: SRTConstructs: Goal setting; action planningSuggested/example intervention component(s): Goal setting and action planning components within the booklet. Template to be completed in discussion with a HCP
**Environmental context and resources**	3.1: Social support (Unspecified) (used and promising) **Definition:** Advise on, arrange or provide social support *(e.g. from friends, relatives, colleagues’, buddies’ or staff)* or non-contingent praise or reward for performance of the behaviour*.* It includes encouragement and counselling, but only when it is directed at the behaviour	Supported by systematic review findings Supported by qualitative findings Expert consensus	Relevant theory: SRT Constructs: Action planning; feedback Suggested/example intervention component(s): Access to repository of information about physical activity options and support via a HCP A booklet showing examples of the physical environment that can be populated (tailored to the individual)
	12.1: Restructuring the physical environment (not used) **Definition:** Change, or advise to change the physical environment in order to facilitate performance of the wanted behaviour or create barriers to the unwanted behaviour (other than prompts/cues, rewards and punishments)	Not used in systematic review, overruled based on strength of qualitative findings Supported by qualitative findings Expert consensus that it is not appropriate for everyone	
**Social influence**	3.1: Social support (Unspecified) **(**used and promising) **Definition:** Advise on, arrange or provide social support *(e.g. from friends, relatives, colleagues’, buddies’ or staff)* or non-contingent praise or reward for performance of the behaviour*.* It includes encouragement and counselling, but only when it is directed at the behaviour	Supported by systematic review findings Supported by qualitative findings Expert consensus	Relevant theory: SRTConstructs: Feedback; action planning; problem solvingSuggested/example intervention component(s): Access to repository of physical activity options and support via a HCP Use of booklet to plan support around physical activity
**Emotion**	3.1: Social support (unspecified**) (**used and promising) **Definition:** Advise on, arrange or provide social support *(e.g. from friends, relatives, colleagues’, buddies’ or staff)* or non-contingent praise or reward for performance of the behaviour*.* It includes encouragement and counselling, but only when it is directed at the behaviour	Supported by systematic review findings Supported by qualitative findings Expert consensus	Relevant theory: SRT Constructs: Feedback; self-monitoring Suggested/example intervention component(s): Access to repository of physical activity options (e.g. local groups) via a HCP to provide ongoing support Use of booklet to monitor effect of PA on emotions and mood followed by feedback from a HCP
	5.4 Monitoring of emotional consequences (not used)	Not used in systematic review, overruled based on strength of qualitative findings Theme from qualitative research Expert consensus	
**Behavioural regulation**	1.1: Goal setting behaviour (used and promising) **Definition:** Def: Set or agree on a goal defined in terms of the behaviour to be achieved	Supported by systematic review findings Supported by qualitative findings Expert consensus	Relevant theory: SRT Constructs: Goal setting; action planning; feedback; self-monitoring Suggested/example intervention component(s): Booklet template to be completed with a HCP. Provision for self-monitoring and feedback (e.g. use of pedometers).
	1.4: Action planning (used and promising) **Definition:** Prompt detailed planning of performance of the behaviour (must include at least one of context, frequency, duration and intensity).	Supported by systematic review findings Supported by qualitative findings Expert consensus	
	2.2: Feedback on behaviour (used and promising) **Definition:** Monitor and provide informative or evaluative feedback on performance of the behaviour *(e.g. form, frequency, duration, intensity)*	Supported by systematic review findings Supported by qualitative findings Expert consensus	
	2.3: Self-monitoring of behaviour (used and non-promising) **Definition:** Establish a method for the person to monitor and record their behaviour(s) as part of a behaviour change strategy	Supported by qualitative findings Expert consensus overruled systematic review. Feedback on behaviour required undertaking of self-monitoring.	

### Theoretical underpinning of the HCP component of the PARAS intervention

Social Cognitive Theory (Bandura, 1986) was selected to underpin the HCP component of the multifaceted intervention. This theory was considered appropriate with reference to the findings of the focus group discussions with HCPs during Step 1. For example, HCPs highlighted the need for specific knowledge and skills development training that would enable them to attain specific practice-related goals. These included promoting and supporting an increase in physical activity to enable improvements in specific functional outcomes of patients. Social Cognitive Theory provides specific examples of evidence-based strategies for translating motivation/intentions into action/behaviour in HCPs through the use of modelling to increase skills and self-efficacy (Godin et al., 2008). Focus group data supported the need for modelling to facilitate skill acquisition and target beliefs about capabilities.

The selection of BCTs incorporated into the HCP component of the intervention was also informed by findings from the qualitative focus group discussions conducted as part of Step 1. The decision-making process is summarised in Table 4 which also describes theoretical constructs targeted and intervention components.

**Table 4.** Theoretical intervention mapping targeting healthcare professional consultation behaviour.

TDF Domain	BCTs identified by healthcare professionals in PARAS qualitative work	Theoretical constructs targeted and potential intervention components
**Knowledge**	5.1: Information about health consequences 9.1: Credible source	Relevant theory: Social Cognitive Theory Constructs: Forethought capability, Vicarious learning capability Suggested/example intervention component(s): A face-to-face training programme presenting research evidence supporting increased physical activity and reduced sedentary behaviour in the context of stroke Case studies of patients who have successfully increased their physical activity levels and/or reduced sedentary time and if possible Case studies from physiotherapists who have successfully supported stroke survivors to be more physically active
**Skills**	1.2: Problem solving 1.4: Action planning 3.1: Social support (practical) 3.3: Social support (emotional) – includes motivational interviewing 4.1: Instruction on how to perform the behaviour (Includes skills training) 5.1: Information about health consequences 6.1: Demonstration of the behaviour	Relevant theory: Social Cognitive Theory Constructs: Forethought capability, Self-regulation capability, vicarious learning capability, Self-efficacy Suggested/example intervention component(s): A manual to accompany the face-to-face training programme which HCPs complete throughout as the training progresses Role play and demonstrations of intervention materials being used Encourage a buddy system among HCPs
**Social/Professional role**	5.2: Salience of consequences	Relevant theory: Social Cognitive Theory Constructs: Outcome expectancies); Forethought, Self-regulation Suggested/example intervention component(s): Verbal delivery explaining the benefits of physical activity promotion and providing ongoing support. Patient narratives
**Beliefs about capabilities**	1.2: Problem solving 1.4: Action planning	Relevant theory: Social Cognitive Theory Constructs: Self-regulation Suggested/example intervention component(s): Problem solving and action planning in the context of practice (i.e. teaching how to complete action planning with patients).
**Beliefs about consequences**	1.2: Problem solving 1.4: Action planning 4.1: Instruction on how to perform the behaviour 6.1: Demonstration of the behaviour	Relevant theory: Social Cognitive Theory Constructs: Forethought, Self-regulation, Vicarious learning Suggested/example intervention component(s): Completion of tasks within the training manual Teaching problem solving and action planning in the context of physiotherapy practice Instruction in the manual on how to action plan and problem solve A demonstration of action planning and problem solving
**Reinforcement**	2.5: Monitoring of outcomes of behaviour without feedback	Relevant theory: Social Cognitive Theory Constructs: Self-regulation Relevant theory: Social Cognitive Theory Constructs: Forethought, Self-regulation, Vicarious learning, Self-efficacy
**Environmental context and resources**	1.2: Problem solving 4.1: Instruction on how to perform the behaviour (includes skills training) 5.2: Salience of consequences 6.1: Demonstration of the behaviour 9.1: Credible source	Suggested/example intervention component(s): Provision of face-to-face training programme with accompanying manual Repository of information providing details of local physical activity groups, support and resources

### Step 4: development of the PARAS intervention

Following the intervention mapping exercise outlined in Step 3, a prototype intervention was developed and presented to stroke survivors and HCPs for feedback during workshops and by questionnaire to inform further iterations.

#### Stroke survivor consultation workshops

We conducted three consultation workshops with stroke survivors (*n* = 21) recruited from local stroke community groups with an aim to elicit views on intervention content, form and mode of delivery. Examples of potential intervention tools (workbook, physical activity diary, information on apps accessible on mobile phone, pedometers) were circulated during workshops to generate discussion and obtain feedback. During the first two workshops (*n* = 13 stroke survivors) a feedback form was used to collate opinions/information (Appendix B). To ensure the intervention could be used by stroke survivors with aphasia (impairment of language), the third workshop was conducted with the North East Aphasia Research User Group (https://www.neta.org.uk/) (*n* = 8 stroke survivors). This workshop was delivered using strategies to enable understanding of language and verbal rather than written feedback was collated. Alongside review of the intervention content and tools, prototype aphasia friendly consent and patient information sheets were reviewed by the group for use during a future feasibility study (Appendix C and D). All three workshops were audio-recorded and transcribed verbatim to facilitate the intervention development processes.

### Key findings of stroke survivor consultation workshops

A detailed overview of workshop findings is provided in Appendix E. In summary, participants reported a preference for the intervention to be supported by HCPs and delivered either at home or in a community outpatient setting. There was a preference for at least two sessions, with the first session delivered face-to-face and subsequent sessions delivered either face-to-face or by telephone. The majority (>75%) of stroke survivors either strongly agreed or agreed that the prototype intervention workbook and physical activity diaries were well organised and easy to use. Eight commercially available pedometers that have been used successfully in other physical activity studies (Carroll et al., 2012; Harris et al., 2017; Sullivan et al., 2014) were presented to stroke survivors during the workshop. The CSX 301S 3D simple pedometer was considered the most appropriate and was the only pedometer to be voted by all participants as easy to use and something they would be likely to use to facilitate self-monitoring.

#### HCP feedback

An online questionnaire was completed by four North East community stroke teams (*n* = 11 HCPs) to elicit feedback on the prototype intervention. These teams had previously expressed an interest in reviewing the intervention and taking part in a future feasibility study.

### Key findings of HCP feedback

Feedback in relation to the intervention design and content was largely positive (Appendix E). Team 2, 3 and 4 strongly agreed or agreed with the suitability of the intervention, tools and mode of delivery. Team 2 were uncertain about whether they could deliver the intervention within their team because they reported discharging patients to other rehabilitation services (i.e. follow-up reviews might not be possible to review goals, provide feedback and discuss problem solving).

Team 1 were concerned about whether their patients would be suitable for the intervention. They perceived the intervention to be for ‘high functioning’ patients who may have been discharged from their service before undertaking the intervention. A further meeting was held with Team 1 to provide more detail that could enable a more informed decision regarding potential participation in a feasibility study of the intervention (e.g. to further emphasise that the intervention was not specifically aimed at ‘high functioning’ patients and could be tailored to individual needs and preferences). Following this meeting, Team 1 agreed they could potentially deliver the intervention.

### Step 5: formulation of an implementation plan

An important consideration for the implementation plan was that it targeted all three pillars of high-quality care: patient experience, safety and effectiveness (Darzi, 2018). To increase the likelihood of implementation, the APEASE criterion: affordability; practicability; effectiveness and cost-effectiveness; acceptability; side effects/safety and equity (Michie, Atkins, & West, 2014) were applied to the intervention design. The final components of the intervention, named Physical Activity Routines After Stroke ‘PARAS’, can be viewed in Table 5. The PARAS intervention targets stroke survivor physical activity and sedentary behaviour via a supported self-management programme and HCP consultation behaviour via a training programme. The stroke survivor and HCP components of the intervention are described in Table 5 using the Template for Intervention Description and Replication (TIDieR) framework. Table 5 also outlines how the APEASE criterion informed selectin of each intervention component.

**Table 5.** PARAS intervention components described with the Template for intervention Description and replication (TIDieR) and APEASE criteria considered in development phase.

TIDieR component	Description	APEASE criteria considered
**Brief name:** Provide the name or a phrase that describes the intervention	Physical Activity Routines After Stroke (PARAS)	
**Why:** Describe any rationale, theory, or gaol of the elements essential to the intervention	See needs assessment step 1–5	
**What**: *materials*: Describe any physical or informational materials used in the intervention, including those provided to participants or used in intervention delivery or in training of intervention providers. Provide information on where the materials can be accessed (e.g. online appendix, URL).	*Components provided to/used by stroke survivors* Consent form and participant information sheet Intervention toolkit including: stroke survivor workbook; repository of local/national information on PA choices; self-monitoring tools (activity diary, pedometer (3DFitBud-Counter-Walking-Pedometer, 3D active, U.K.) and instructions, app advice); laminated goal summary sheet and fridge magnet pen; laminated benefits, outcomes and activities cards to aid discussion between stroke survivor and HCP and support people with speech and language problems *Components provided to/used by healthcare professionals* Consent form and participant information sheet HCP training brochure Dictaphone	*Affordability:* Portable document format (PDF) files of all the intervention tools were created, printed out and stored in a workbook file meaning extra patient specific sheets could be added to individual’s files (e.g. physical activity diary). This process allowed iterative changes to be made without large costs of reprinting manuals. Rather than creating a website with large costs linked to maintenance and development, it was decided to trial a paper-based version of the intervention initially which could be developed online at a later date. The pedometer selected had a relatively low price point (£16.99) to enable increased sued with NHS settings. *Practicability*: HCPs were provided with a PARAS kitbag holding all the intervention tools so they could deliver the intervention then and there rather than having to for example find out information about available resources and get back to participants at a later date. *Acceptability:* all components tested at co-design workshops and developed iteratively in response to feedback *Equity:* The stroke survivor intervention tools were designed to be inclusive so individuals with speech and language or cognitive difficulties would not be excluded as is the case in the majority of stroke research studies.
**What:***procedures*: Describe each of the procedures, activities, and/or processes used in the intervention, including any enabling or support activities.	*Stroke survivor procedures* Supported self-management programme involving goal setting, action planning, barrier identification, coping planning and feedback around PA and sedentary behaviour. *HCP procedures* Training programme aimed at developing physical/sedentary behaviour counselling skills of HCPs. Initial training before delivery of intervention then feedback provided on delivery.	*Acceptability, affordability and practicability*: Supported self-management was identified as the most appropriate mode of delivery for the stroke survivor component following our needs assessment and co-design workshops. This type of intervention appears more sustainable than for example face-to-face structured group exercise which presents with a number of environmental and resource related barriers. *Acceptability and effectiveness:* Although HCPs working in community stroke care will have some experience of goal setting etc. qualitative workshops identified there were training needs in this area and it was acceptable to target these needs.
**Who:** For each category of intervention provider (e.g. psychologist, nursing assistant), describe their expertise, background and any specific training given.	*Provider of stroke survivor component* A healthcare professional (HCP) who is a credible source (e.g. well informed on stroke rehabilitation) and plays a key role in the stroke survivors community rehabilitation e.g. physiotherapist, occupational therapist, nurse. *Provider of HCP component* Health psychologist with experience in delivering behaviour change interventions in long-term conditions, research physiotherapist with 20 years clinical experience and 10 years research experience in developing and delivery physical activity and rehabilitation stroke interventions	*Affordability, practicability and acceptability*: PARAS focus groups identified delivery of the stroke survivor component should be by a healthcare professional with experience working in stroke. Using healthcare professionals embedded within community stroke teams meant these individuals already had specialist core stroke skills meaning training was not required in this area alongside training in PARAS delivery. As the intervention was designed to be delivered within usual care this meant there were not additional salary costs. Initially consideration was made to include technical instructors and rehab assistants however on discussion with these individuals it was felt they would prefer to support the delivery rather than lead on the delivery and that they were not happy to be audio-recorded as part of the fidelity assessment. *Acceptability and effectiveness*: As the providers of the HCP training had developed the intervention and were experienced in this field from both a therapy and a psychology perspective they were thought to be the most credible source to deliver the training. *Practicability:* At this feasibility stage it was decided that two members of the research team would deliver the HCP training face-to-face. This allowed the research team to highlight any iterative changes required to the training programme before scaling.
**How:** Describe the modes of delivery (e.g. face-to-face or by some other mechanism, such as internet or telephone) of the intervention and whether it was provided individually or in a group	*Stroke survivor component* First session face-to-face, follow-up sessions either face-to-face or remotely by phone dependent on patient choice. *HCP training component* Face-to-face for initial training, then email and phone contact to provide feedback	Acceptability: The modes of delivery were assessed as acceptable from our needs assessment, co-design workshops and questionnaires. *Affordability and practicality*: Our qualitative work indicted that this mode of delivery of the stroke survivor intervention was practical. As the community HCP involved in delivering the stroke survivor component were already working with the stroke survivors participating in the study it was practical for them to initially see the participants face-to-face. To lower travel costs the option of providing the review sessions by phone was provided *Acceptability, affordability and practicality*: The HCPs were very clear that they wanted training face-to-face as they felt E-Learning was not effective. The HCP component was delivered in a group format to each of three community teams involved. This reduced costs of training individuals. Delivering three spate sessions rather than delivering the training to everyone at once meant that the training could be fitted around the needs of each service.
**Where:** Describe the type(s) of location(s) where the intervention occurred, including any necessary infrastructure or relevant features.	*Stroke survivor component* UK NHS community stroke services. Delivered In patient’s homes or outpatient settings *HCP training component* Initial face-to-face training delivered at participating community stroke teams’ education centres.	*Acceptability:* Our needs assessment, co-design workshops and resource capacity mapping exercise within services identified the acceptability of the intervention location. *Practicality:* It was hypothesised that delivering the stroke survivor intervention within patients homes would allow the HCP to provide better support to the participants as they would have an increased understanding of the participants environmental circumstances. This was felt to outweigh costs associated with community visits. *Affordability:* Although travelling to the community sites to deliver the HCP training had cost implications, it was believed more HCP would attend if the trainers travelled to the participants. In the future, it is thought that this training could be delivered online but at this stage to enable as much knowledge on delivery face-to-face training was deemed most appropriate.
**When and how much:** Describe the number of times the intervention was delivered and over what period of time including the number of sessions, their schedule, and their duration, intensity or dose.	*Stroke survivor component* At least two sessions. The first session/s targets goal setting using the workbook and other tools, there will then be a review session timed to coincide with review date for PA/sedentary behaviour goals. There is no upper limit to sessions the number is defined by patients’ support needs/availability of resources. The programme to be initiated once ‘functional rehab’ goals have been achieved and the stroke survivor is moving towards supported self-management. The time from stroke will vary dependent on needs of participant/health care professional’s opinion on the best timing/availability of resources. *HCP training component* Three-hour face-to-face training session. Email and phone delivery support by study team. Email feedback on the delivery of the intervention after completion with two stroke survivors.	*Acceptability:* Our needs assessment and co-design workshops identified what was acceptable in terms of timing and dose of delivery of the components of the intervention. *Side effects/safety:* Our needs assessment and co-design workshops provided evidence that the intervention training and delivery methods would be safe with minimal side effects. As the stroke survivors were already being seen by a community stroke team with specialist skills it was felt that this team would be able to effectively identify any risks associated with taking part in the intervention and potential changes in physical/sedentary behaviour. *Effectiveness*: It was hypothesised that all the components of the stroke survivor intervention could be delivered effectively within the two or more sessions. It was also hypothesised that all elements of the stroke survivor intervention delivery could be taught effectively within three hours with email and phone contact for support during delivery *Affordability*: The supported self-management approach for the stroke survivor component provided a more affordable but at the same time potentially effective method of delivery than for example a face-to-face exercise intervention.
**Tailoring:** If the intervention was planned to be personalised, titrated or adapted, then describe what, why, when, and how	*Stroke survivor component* Support graded to individuals’ ability, preference, and values and progressed as able *HCP training component* The HCP training was tailored according to personal needs during the face-to-face training, email and telephone support. All participating HCPs received feedback on delivery that was tailored to their individual learning needs.	*Acceptability:* The needs assessment, co-design workshops and questionnaires were used to assess the acceptability of tailoring. *Affordability:* To enable effectiveness our needs assessment identified that a person centred individual tailored approach was required for the stroke survivors. This approach is potentially more expensive than a group based approach, however the increased potential for effectiveness should outweigh these costs. *Equity:* the stroke survivor component was designed to allow a person-centred tailored approach that would not exclude any stroke survivor who has the potential to move more or sit less.
**How well:** Planned: If intervention adherence or fidelity was assessed, describe how and by whom, and if any strategies were used to maintain or improve fidelity, describe them.	Treatment fidelity strategies for design of study HCP training. Plan for implementation setbacks e.g. map out stroke team resources prior and during study and plan in case anyone is leaving, rotating etc. Treatment fidelity strategies for monitoring and improving provider training Face to face training and standardised training manual provided to HCP. Testing of HCP acquisition skills during training and delivery. Minimise drift in HCP skills during programme delivery e.g. monitor how work books are completed and delivery of sessions through audio recording and checklist completion. Programme leads available to provide training on aspects of delivery on request. Tailor training to needs of HCPs delivering programme Treatment fidelity strategies for monitoring and improving delivery of programme Assessment of delivery of programme through audio recording and analysis of sessions and completion of workbook to enable provision of feedback and training on delivery of programme to HCPs Treatment fidelity strategies for monitoring and improving receipt of programme Assess participants understanding of programme, use of cognitive skills and ability to perform behavioural skills through completion of workbook and analysis of audio-recorded sessions Treatment fidelity strategies for monitoring and improving enactment of programme skills Review workbook completion and achievement of goals.	*Practicability and effectiveness*: Informed by previous delivery of physical activity interventions in diabetes and cardiovascular disease. Fidelity assessment based upon Bellg, A. J., Borrelli, B., Resnick, B., Hecht, J., Minicucci, D. S., Ory, M., & Czajkowski, S. (2004). Enhancing treatment fidelity in health behavior change studies: best practices and recommendations from the NIH Behavior Change Consortium. *Health Psychology, 23*(5), 443. *Acceptability:* The main issue brought up by the HCPs during the devlopment phase was the need to audio-record the intervention delivery. When it was discussed that this approach was to detrmine whether our training programme was appropaite the HCPs stated they thought this was acceptable. Whether this was actually the case will be further tested in a feasbility study.

### Step 6: development of an evaluation plan

To further develop and optimise the intervention within community stroke settings, in accordance with the MRC framework, the next step was to develop an intervention evaluation plan. Therefore, a protocol for a study determining the feasibility of the PARAS intervention, was developed (Moore et al., 2020). The feasibility study is registered on the ISRCTN website (Trial identifier: ISRCTN35516780, date of registration: 24/10/2018 URL http://www.isrctn.com/ISRCTN35516780).

## Discussion

Low levels of physical activity and high levels of sedentary behaviour are common following stroke (Moore et al., 2013) and are associated with cardiovascular health, mental health and quality of life (Moore et al., 2014). An intervention development process, informed by the MRC guidelines for the development and evaluation of complex interventions, (Craig et al., 2013) using Intervention Mapping as a framework (Bartholomew et al., 2011) was undertaken to target physical activity and sedentary behaviour in the context of stroke rehabilitation as part of the routine care pathway in acknowledgement that stroke survivors have specific information and support requirements. An initial needs assessment identified a lack of effective theory-and-evidence informed interventions targeting long-term free-living physical and sedentary behaviour in stroke survivors (Moore et al., 2018) and our qualitative work identified the need to develop a timely, sustainable person-centred supported self-management intervention to address this problem.

Historically, structured exercise has been the most common mode of targeting physical activity after stroke (Saunders et al., 2020). Although structured exercise can lead to short-term changes in function, how this mode of delivery impacts on long-term health and well-being has not been established. Perhaps more importantly, our qualitative research mirrored previous findings indicating that many barriers exist to this approach in terms of implementation e.g. resources, training, access and costs that make it unsustainable for many stroke survivors (Nicholson et al., 2012). Furthermore, structured exercise does not account for individual physical activity needs and preferences. Our qualitative work indicated that stroke survivors wish to partake in activities that provide meaning to their lives and allow them to recapture activities they engaged in prior to experiencing a stroke. This may be through structured exercise, but more commonly reported was engagement in day-to-day activities such as washing the car, shopping, or playing with grandchildren. This finding supports previous qualitative work undertaken in stroke (Nicholson et al., 2012). It was, therefore, important that the intervention developed was person-centred rather than ‘one size fits all’. The implementation mapping process led to the development of a supported self-management intervention delivered at an appropriate time point within the rehabilitation pathway, tailored to individual needs with training for HCPs delivering the intervention. This training was designed to provide HCPs with specific evidence-informed education about physical activity and sedentary behaviour in the context of stroke, and competency in using BCTs to target problem solving for example.

There is an expectation within self-management in stroke and at a governmental level that person-held experience is incorporated into healthcare intervention design (Kulnik et al., 2019). Early engagement with stroke survivors and HCPs during the intervention development process was a key strength of this study, which can potentially enable future implementation, with those taking part in the process becoming champions for the intervention (Clarke et al., 2017). Aphasia is a common communication problem affecting approximately one-third of stroke survivors (Engelter et al., 2006). Engaging with patients with aphasia during co-design is complex and as a result is often not undertaken leading to interventions that are inappropriate for this population and leaving a sub-population unsupported. In previous self-management interventions in stroke, up to 46% of studies have excluded individuals with aphasia limiting extrapolation of findings to large numbers of stroke survivors (Brady, Fredrick, & Williams, 2013). One of the strengths of our intervention development process which may enable implementation was engagement with a group of stroke survivors with aphasia, and the incorporation of their views into the intervention design.

Alongside the incorporation of user views, another strength of the study was the use of the APEASE criteria to consider the social context of intervention delivery to facilitate implementation (Michie et al., 2014). Our systematic review highlighted that the majority of RCTs and pilot studies in this field have been led by research teams, not clinicians, and attempts have not been made to embed testing within existing clinical pathways and settings (English et al., 2016; Jones et al., 2016; Moore et al., 2018; Preston et al., 2017). The PARAS intervention was developed with implementation into the clinical care pathway in mind, because implementation of research findings into rehabilitation settings has demonstrated previously to be slow, with evidence often not influencing practice (Morris et al., 2020).

Our needs assessment highlighted that the intervention should be multi-faceted, targeting both the behaviour of the stroke survivors and the behaviour of the HCPs providing support. This is a novel approach in this field, where most interventions focus exclusively on stroke survivors and those providing interventions being expected to do so without training or support. Our qualitative work indicated that stroke survivors have a preference to be supported by a HCP, therefore it was important to consider behavioural change counselling strategies for use by HCPs to enable this. It could be argued that HCPs already have skills to support these long-term behavioural changes, however, observational data on habitual physical activity and sedentary behaviour post-stroke (Fini et al., 2017) indicates the contrary and the findings of our qualitative study highlighted the need for training in this area. Previous research further suggests that perceptions on physical activity post-stroke vary between stroke survivors, informal carers and HCPs, (Morris et al., 2015) therefore training on how to deliver person-centred support to enable meaningful engagement in physical activity, which is more likely to result in long-term change, may be required.

It is anticipated that the application of complex intervention development processes will increase the likelihood of future effectiveness and implementation of the intervention. However, several limitations associated with our developmental process should be acknowledged. Stroke survivors that took part in the initial qualitative focus group discussions were required to travel, meaning only those who had access to transport or were mobile could attend. In addition, invitations to participants in these groups were advertised mainly at local stroke groups or patient carer panels which may have limited representation of a general stroke population. Although we advertised for informal carers to attend the focus groups, only three took part and information contributed was minimal and did not enable formal analyses. Therefore, this limited our understanding from a carer perspective. The HCPs recruited may not have been representative as they were self-selected limiting generalisability. The majority were physiotherapists and assistants, rather than from the broad range of disciplines who may also have been suitable to deliver the intervention e.g. nurses, occupational therapists, speech and language therapists, exercise on referral/fitness instructors.

## Conclusions

Effectively targeting complex behaviours such as physical activity and sedentary behaviour post-stroke requires systematic and iterative development of evidence and theory informed interventions. Alongside effectiveness, the likelihood of adoption, implementation and sustainability of an intervention should also be considered during the development process. We have presented the development of an intervention, grounded in stroke survivor and service provider perspectives that targets long-term habitual physical activity and sedentary behaviour post-stroke. Throughout the developmental process, there was active engagement of stroke survivors and HCPs to increase likelihood of the acceptability and effectiveness of the intervention and long-term implementation. The PARAS intervention is currently being testing in three North East community stroke services and the results of this feasibility study will further inform development. Following the MRC guidelines for the development and evaluation of complex interventions, the intervention will be further evaluated assessing efficacy, cost-effectiveness and process.

## Supplementary Material

Supplemental Material

## Data Availability

The datasets used and/or analysed during the current study are available from the corresponding author on reasonable request.
