# Supplementary material for: Using intervention mapping to develop and facilitate implementation of a multifaceted behavioural intervention targeting physical activity and sedentary behaviour in stroke survivors: Physical Activity Routines After Stroke (PARAS): intervention development study
Source: Health Psychol Behav Med. 2022 May 12;10(1):439–66. doi: 10.1080/21642850.2022.2066534 (PMC9116237; doi:10.1080/21642850.2022.2066534)
Supplement: Supplemental Material [file RHPB_A_2066534_SM6283.docx]

**Supplementary tables**

**Appendix A: Service and capacity mapping community stroke teams**

|  | Community stroke service 1 | Community stroke service 2 | Community stroke service 3 |
| --- | --- | --- | --- |
| Type of therapy service | Early supported discharge service (delivered at home) | Early supported discharge service (delivered at home) | Early supported discharge service (home) and community neurology (outpatient or home) |
| Potential staffing available for project  Physiotherapists (Band 5-7)  Technical instructors (TI) Band 4  Occupational therapists (Band 5-7)  Rehab/support workers | 3  2  2 | 2  3  3 | 3 (2 physios 0.5 WTE).  1  1  6 part time |
| Rotational /static posts | Static | Static | Static/rotational |
| Likelihood of staff changes during study | Unlikely. Band 4 TI may change before project starts but will then be permanent | Unlikely | **Band 7 retiring 2019  Band 6 six month rotational post |
| Do you see all patients who have had a stroke | No some are discharged straight from inpatients | Yes | No |
| Patient referral rate per month | 32 per month | 18-20 per month | 10 per month |
| Approximate amount of face-to-face contact with patients | Dependent on patient needs | Dependent on patient needs | Dependent on patient needs |
| Is the service time limited | no | no | No |
| Are patients referred onto other physio services | no | Occasionally to falls service, neurology outpatients | Approximately 10% of patients referred from early supported discharge to community neurology |
| Do you have a stroke review clinic | Yes consultant based at 6 weeks and a nurse led clinic at 6 months | Yes 6 weeks and six months | Yes 6 months run by nurse practitioner/consultant |
| Do you have any local guidelines/procedures about supporting physical activity and reducing sedentary behaviour after stroke | Starting to develop some guidelines and materials to encourage exercise programme uptake with support of carers on stroke unit. This is currently not happening in the community | Patients receive general stroke information pack | No specific guidelines/procedures for supporting physical activity |

**Appendix B Stroke survivor/informal carer co-design workshop feedback form**

**Workbook feedback**

Please indicate your response by ticking one of the boxes using the scoring system below:

**Scoring: 1 = Strongly Disagree; 2 = Disagree; 3 = Undecided; 4 = Agree; 5 = Strongly Agree**

**Benefits of exercise**

|  | **Benefits of exercise** |
| --- | --- |
| The planned content is well organised | **1□ 2 □ 3 □ 4□ 5□** |
| The planned content is easy to follow | **1□ 2 □ 3 □ 4□ 5□** |
| The planned design is appropriate | **1□ 2 □ 3 □ 4□ 5□** |

Comments: …………………………………………………………………………………………………………………………………………..……………

**Outcomes**

|  | **Planned activities** |
| --- | --- |
| The planned content is well organised | **1□ 2 □ 3 □ 4□ 5□** |
| The planned content is easy to follow | **1□ 2 □ 3 □ 4□ 5□** |
| The planned design is appropriate | **1□ 2 □ 3 □ 4□ 5□** |
| There are no outcomes missing | **1□ 2 □ 3 □ 4□ 5□** |

Comments: …………………………………………………………………………………………………………………………………………………………

**Planned activities**

|  | **Planned activities** |
| --- | --- |
| The planned content is well organised | **1□ 2 □ 3 □ 4□ 5□** |
| The planned content is easy to follow | **1□ 2 □ 3 □ 4□ 5□** |
| The planned design is appropriate | **1□ 2 □ 3 □ 4□ 5□** |
| There are no planned activities missing | **1□ 2 □ 3 □ 4□ 5□** |

Comments: …………………………………………………………………………………………………………………………………………..……………

**Goal setting and action plans**

|  | **Goal setting and progress review** |
| --- | --- |
| The planned content is well organised | **1□ 2 □ 3 □ 4□ 5□** |
| The planned content is easy to follow | **1□ 2 □ 3 □ 4□ 5□** |
| The planned design is appropriate | **1□ 2 □ 3 □ 4□ 5□** |
| The example goal sheets are useful | **1□ 2 □ 3 □ 4□ 5□** |

Comments: …………………………………………………………………………………………………………………………………………..……………

**Barrier identification**

|  | **Goal setting and progress review** |
| --- | --- |
| The planned content is well organised | **1□ 2 □ 3 □ 4□ 5□** |
| The planned content is easy to follow | **1□ 2 □ 3 □ 4□ 5□** |
| The planned design is appropriate | **1□ 2 □ 3 □ 4□ 5□** |

Comments: …………………………………………………………………………………………………………………………………………..……………

**Reviewing progress and future plans**

|  | **Goal setting and progress review** |
| --- | --- |
| The planned content is well organised | **1□ 2 □ 3 □ 4□ 5□** |
| The planned content is easy to follow | **1□ 2 □ 3 □ 4□ 5□** |
| The planned design is appropriate | **1□ 2 □ 3 □ 4□ 5□** |

Comments: …………………………………………………………………………………………………………………………………………..……………

**General instructions**

|  | **General instructions** |
| --- | --- |
| The planned content is well organised | **1□ 2 □ 3 □ 4□ 5□** |
| The planned content is easy to follow | **1□ 2 □ 3 □ 4□ 5□** |
| The planned design is appropriate | **1□ 2 □ 3 □ 4□ 5□** |

Comments: …………………………………………………………………………………………………………………………………………..……………

**Overall views of workbook**

|  | **Introduction** |
| --- | --- |
| The planned content is well organised | **1□ 2 □ 3 □ 4□ 5□** |
| The planned content is easy to follow | **1□ 2 □ 3 □ 4□ 5□** |
| The planned design is appropriate | **1□ 2 □ 3 □ 4□ 5□** |
| There is the right amount of information | **1□ 2 □ 3 □ 4□ 5□** |

Comments: …………………………………………………………………………………………………………………………………………..……………

**Physical activity diaries**

**Scoring: 1 = Strongly Disagree; 2 = Disagree; 3 = Undecided; 4 = Agree; 5 = Strongly Agree**

**Activity tracker**

|  | **Activity tracker** |
| --- | --- |
| The physical activity diary would be helpful | **1□ 2 □ 3 □ 4□ 5□** |
| The content of the physical activity diary is correct | **1□ 2 □ 3 □ 4□ 5□** |

Comments: …………………………………………………………………………………………………………………………………………..

**Apps**

Below are some examples of apps that are recommended for supporting physical activity after stroke:

**Couch to 5K (running app) SitFit (seated exercise app) Human Activity tracker (measures walks, runs and bike rides)**

[
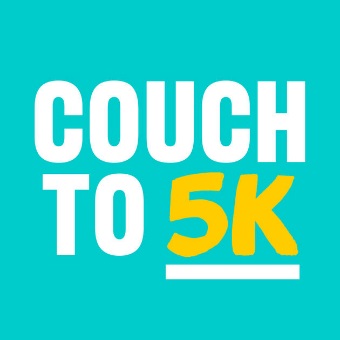
](https://www.google.co.uk/url?sa=i&rct=j&q=&esrc=s&source=images&cd=&cad=rja&uact=8&ved=0ahUKEwiC5fOhy4fZAhXpA8AKHcXvBgkQjRwIBw&url=https://itunes.apple.com/gb/app/one-you-couch-to-5k/id1082307672?mt%3D8&psig=AOvVaw2W7Xa80UNmQDSwnQJ8QnKX&ust=1517673165135535) [
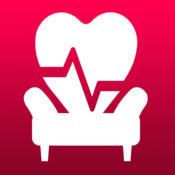
](https://www.google.co.uk/url?sa=i&rct=j&q=&esrc=s&source=images&cd=&cad=rja&uact=8&ved=0ahUKEwjW3fi1y4fZAhWqI8AKHVf1AesQjRwIBw&url=https://itunes.apple.com/us/app/sitfit-exercise/id1083935773?mt%3D8&psig=AOvVaw1uUyT-1LUBLlBEIj06lmgH&ust=1517673207932196) [
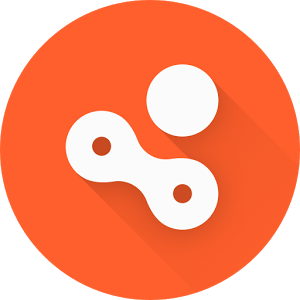
](https://www.google.co.uk/url?sa=i&rct=j&q=&esrc=s&source=images&cd=&cad=rja&uact=8&ved=0ahUKEwj6oJ2DzIfZAhVJL8AKHdtNBBcQjRwIBw&url=https://play.google.com/store/apps/details?id%3Dco.human.android&psig=AOvVaw2a6kpQW34-EbWFz1FdTnhz&ust=1517673368752410)

**Map my walk Simple workout log**

**(Measures walks and tracks progress)**

[
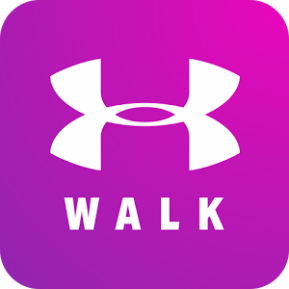
](https://www.google.co.uk/url?sa=i&rct=j&q=&esrc=s&source=images&cd=&cad=rja&uact=8&ved=0ahUKEwitjvLDzIfZAhXLLMAKHY9rDMQQjRwIBw&url=https://play.google.com/store/apps/details?id%3Dcom.mapmywalk.android2%26hl%3Den_GB&psig=AOvVaw3yD1fLUopj4sVpYQWS1PT0&ust=1517673502438529) [
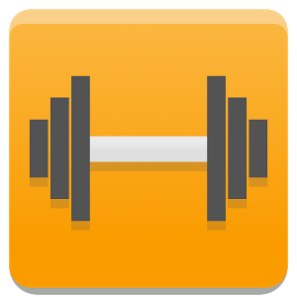
](https://www.google.co.uk/url?sa=i&rct=j&q=&esrc=s&source=images&cd=&cad=rja&uact=8&ved=0ahUKEwjGtvL2zIfZAhXlIMAKHVyPBG4QjRwIBw&url=https://play.google.com/store/apps/details?id%3Dcom.selahsoft.workoutlog&psig=AOvVaw1QvZ5LGULZvs-st0V9tvmU&ust=1517673611483549)

Apps

|  | **Apps** |
| --- | --- |
| My phone has the capacity to download apps | **1□ 2 □ 3 □ 4□ 5□** |
| I download apps onto my phone regularly | **1□ 2 □ 3 □ 4□ 5□** |
| The apps above would be suitable for people with stroke | **1□ 2 □ 3 □ 4□ 5□** |
| I would use an app to support my physical activity | **1□ 2 □ 3 □ 4□ 5□** |

Have you used any apps to monitor your physical activity: Yes/No

If yes which apps have you used?

Did you find them useful?

Comments: …………………………………………………………………………………………………………………………………………..……………

**Pedometers**

**Scoring: 1 = Strongly Disagree; 2 = Disagree; 3 = Undecided; 4 = Agree; 5 = Strongly Agree**

**OMRON Walking style One 2.1**

|  | **OMRON** |
| --- | --- |
| This pedometer is easy to use | **1□ 2 □ 3 □ 4□ 5□** |
| I would use this pedometer to track my daily steps | **1□ 2 □ 3 □ 4□ 5□** |

Comments: …………………………………………………………………………………………………………………………………………..

**3D TRI-FIT**

|  | **3D Tri-Fit** |
| --- | --- |
| This pedometer is easy to use | **1□ 2 □ 3 □ 4□ 5□** |
| I would use this pedometer to track my daily steps | **1□ 2 □ 3 □ 4□ 5□** |

Comments: …………………………………………………………………………………………………………………………………………..

**Scoring: 1 = Strongly Disagree; 2 = Disagree; 3 = Undecided; 4 = Agree; 5 = Strongly Agree**

**YAMAX DIGI-WALKER**

|  | **YAMAX DIGI-WALKER** |
| --- | --- |
| This pedometer is easy to use | **1□ 2 □ 3 □ 4□ 5□** |
| I would use this pedometer to track my daily steps | **1□ 2 □ 3 □ 4□ 5□** |

Comments: …………………………………………………………………………………………………………………………………………..……………

**CSX: Competitive Sport Xtreme**

|  | **CSX** |
| --- | --- |
| This pedometer is easy to use | **1□ 2 □ 3 □ 4□ 5□** |
| I would use this pedometer to track my daily steps | **1□ 2 □ 3 □ 4□ 5□** |

Comments: …………………………………………………………………………………………………………………………………………..……………

**Scoring: 1 = Strongly Disagree; 2 = Disagree; 3 = Undecided; 4 = Agree; 5 = Strongly Agree**

**Mountain Warehouse**

|  | **Mountain Warehouse** |
| --- | --- |
| This pedometer is easy to use | **1□ 2 □ 3 □ 4□ 5□** |
| I would use this pedometer to track my daily steps | **1□ 2 □ 3 □ 4□ 5□** |

Comments: …………………………………………………………………………………………………………………………………………..……………

**3D TriSport**

|  | **3D TriSport** |
| --- | --- |
| This pedometer is easy to use | **1□ 2 □ 3 □ 4□ 5□** |
| I would use this pedometer to track my daily steps | **1□ 2 □ 3 □ 4□ 5□** |

Comments: …………………………………………………………………………………………………………………………………………..

**Scoring: 1 = Strongly Disagree; 2 = Disagree; 3 = Undecided; 4 = Agree; 5 = Strongly Agree**

**PINGKO**

|  | **PINGKO** |
| --- | --- |
| This pedometer is easy to use | **1□ 2 □ 3 □ 4□ 5□** |
| I would use this pedometer to track my daily steps | **1□ 2 □ 3 □ 4□ 5□** |

Comments: …………………………………………………………………………………………………………………………………………..

**SPORTLINE 330**

|  | **PINGKO** |
| --- | --- |
| This pedometer is easy to use | **1□ 2 □ 3 □ 4□ 5□** |
| I would use this pedometer to track my daily steps | **1□ 2 □ 3 □ 4□ 5□** |

Comments: …………………………………………………………………………………………………………………………………………..

**Pedometer**

|  | **Pedometer** |
| --- | --- |
| Would you use a pedometer to support your physical activity? | **1□ 2 □ 3 □ 4□ 5□** |

Which pedometer did you like the best? Why?

Comments: …………………………………………………………………………………………………………………………………………..

**Discussion points**

**Who do you think should support stroke patients to use the workbook and undertake the intervention?**

**How do you think you think progress should be reviewed to help people maintain physical activity habits e.g. face to face sessions, phone calls email?**

**How often should progress be reviewed to help people maintain physical activity habits?**

**Where do you think the intervention should be delivered?**

**Do you think there are any more tools that might help people to start and maintain physical activity after stroke?**

**How do you think the outcome of the intervention should be measured?**

**Appendix C Aphasia friendly consent form**

**Consent form for Stroke Survivors**

**Taking part in research about stroke and physical activity**

**PARAS Physical Activity Routines After Stroke**


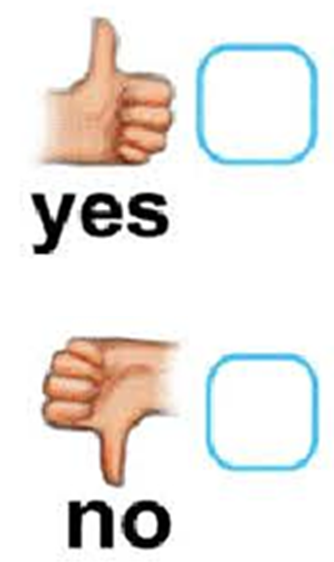


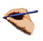


Please **mark** for each statement

**To be read aloud for people who cannot read**


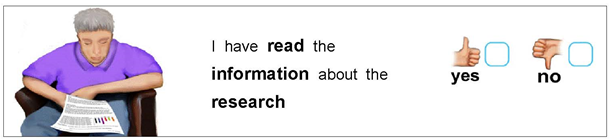


I have been **given** the **information** about the **research**

**dated 31st August 2018**

**I have read the information**


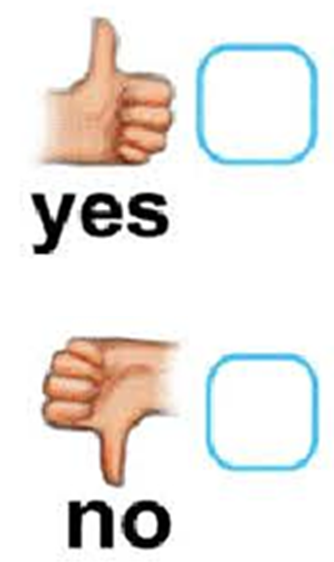


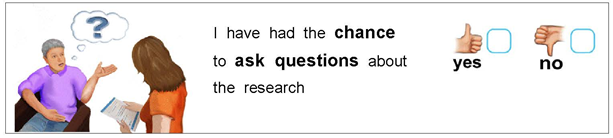


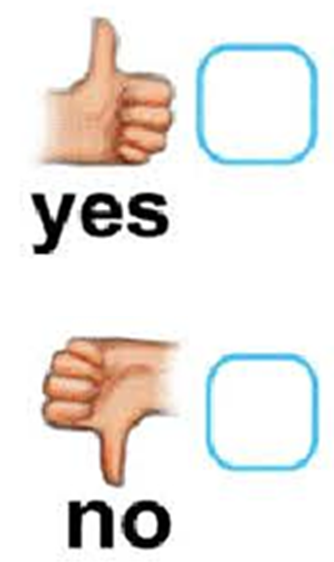


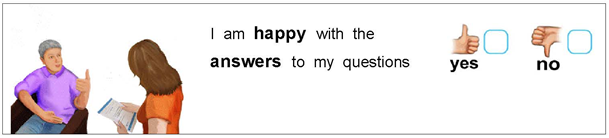


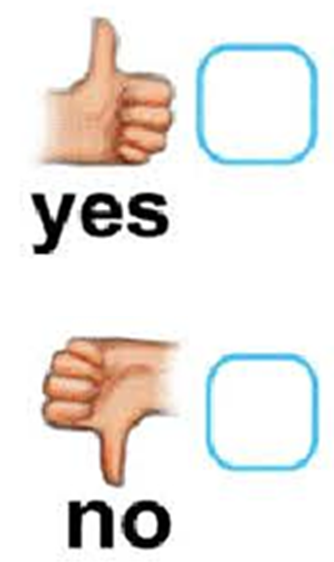


I understand that the therapist will make

**sound recordings of what we say to each other**


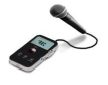


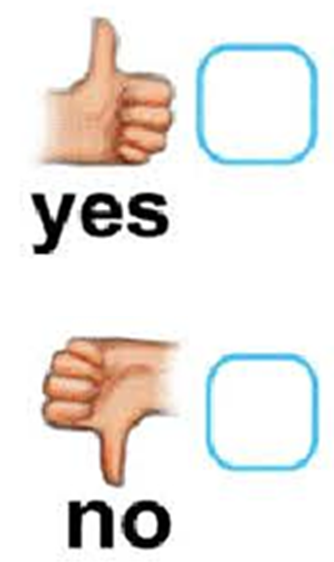


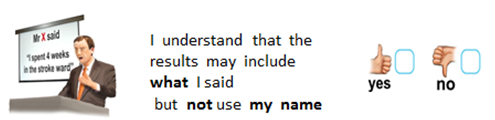


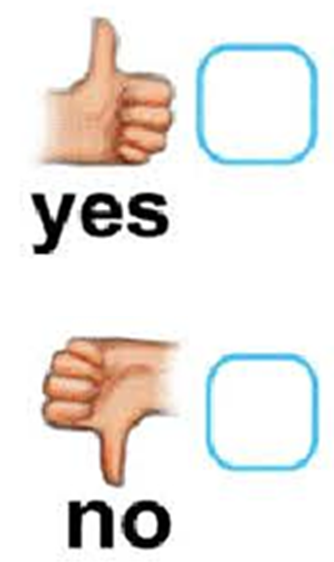


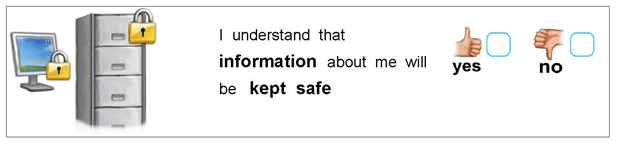


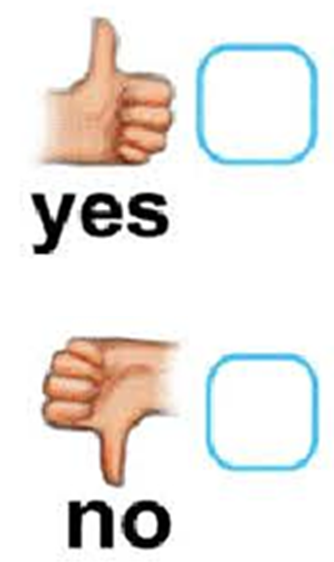


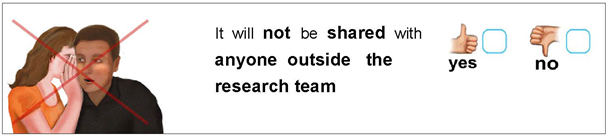


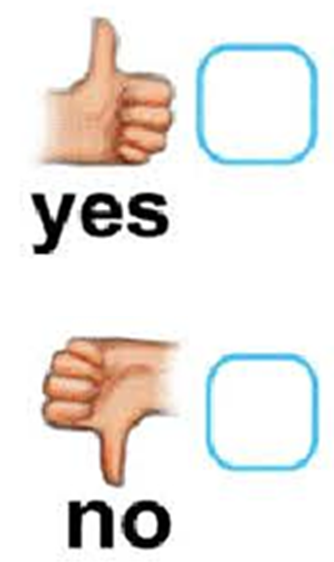


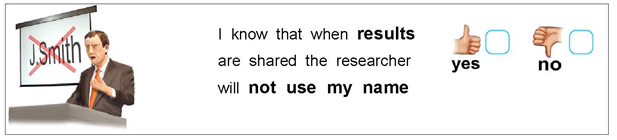


I understand that **I can stop** **being in the research** at any time

**If I stop I do not** have to **give a reason**

And I will **still get my normal therapy**

Any **information already collected** about you will **still be used** **unless you ask** for this **not to happen**


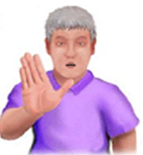


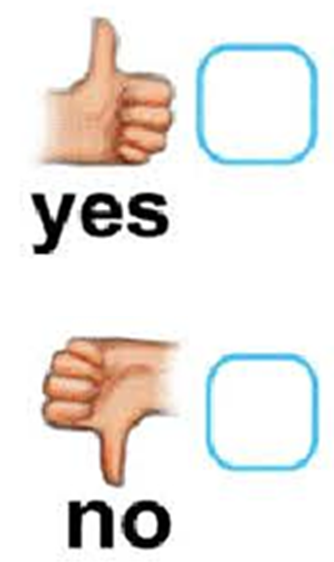


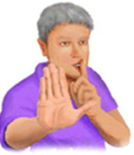


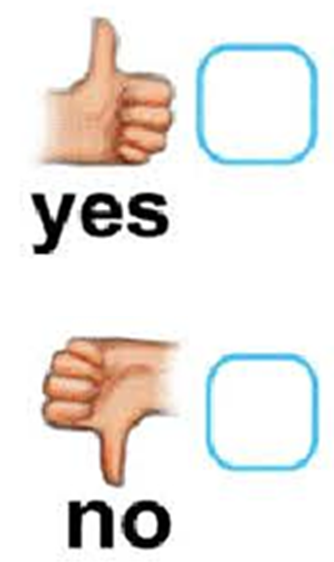


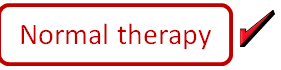

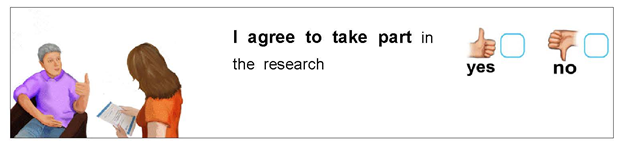

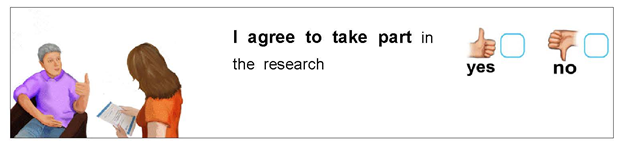


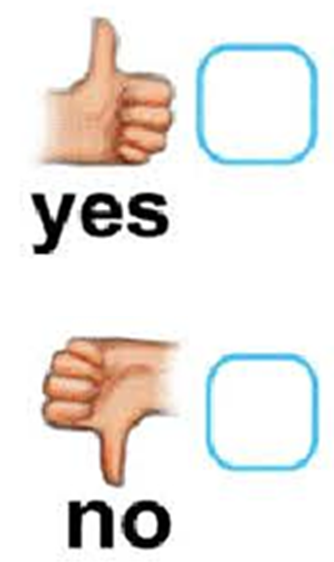

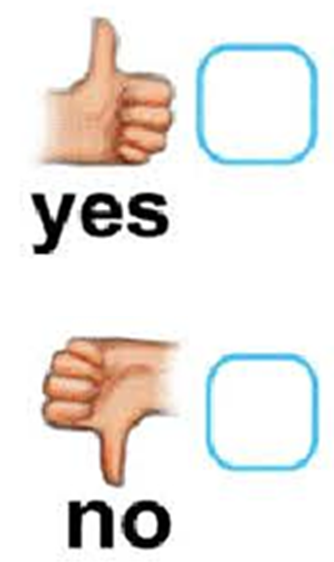


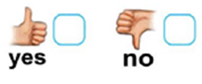


**I would like** the **results**

of the research


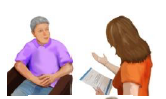


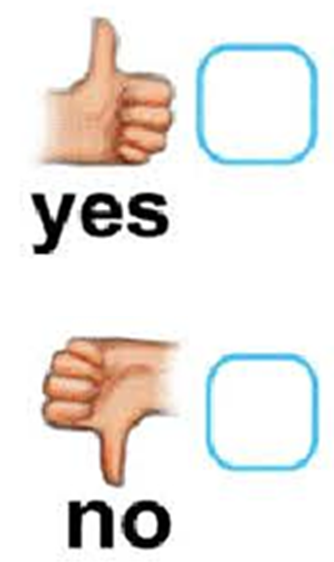


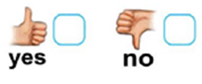


**If I agree to take part**

I would be **happy** to have

an **interview**

to **share** my **experiences** and **opinions** about the research


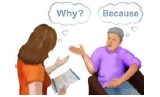


**I agree** that my **GP** will be **told** that I am **in this study**


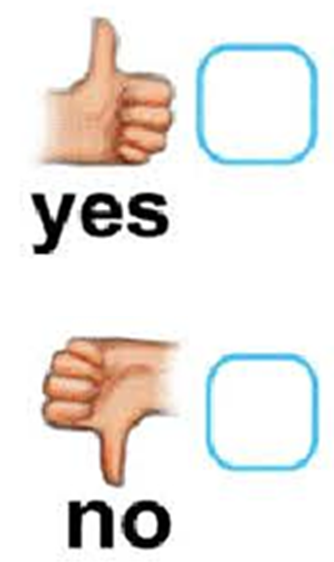


**I give my consent to take part in this study:**


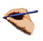


**Name of Stroke Survivor_________________________________**


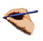


**Signature of Stroke Survivor______________________________**


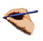
 **Name and Signature of witness (if stroke survivor cannot sign e.g. due to hand weakness after stroke)**

**______________________________________________________________**


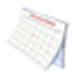


**Date__________________**

**Details of person taking consent:**

**Name___________________ Signature _______________ Date______**


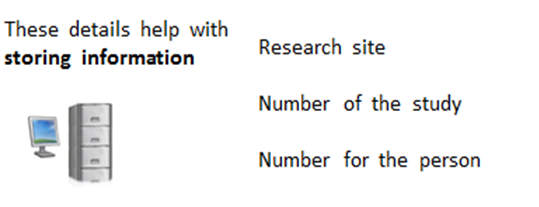


**Appendix D Aphasia friendly information sheet**

**
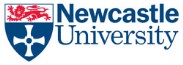
** *[insert Trust logo here]*

**Information Sheet for Stroke Survivors and Carers**

**What is the PARAS research project?**

| 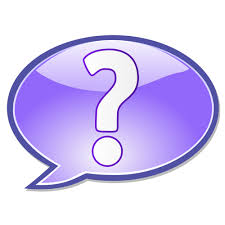  **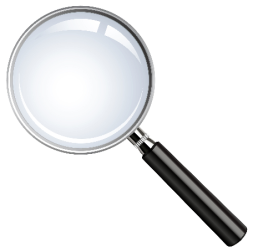** | The **PARAS** research project is for people who have had a **stroke**  Research helps us **learn**  We want you to **think about** taking part |
| --- | --- |

**Why are we doing the research?**

| 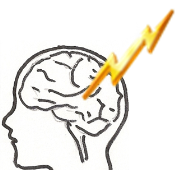   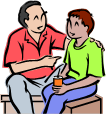 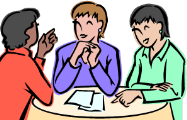 | Just after a **stroke**  **Therapy** is given to make things better **(rehabilitation)** |
| --- | --- |
| **** | It is important to **keep active,** **move around and stop sitting** after stroke  This **helps therapy (rehabilitation)**  This **helps general health** |
| ****  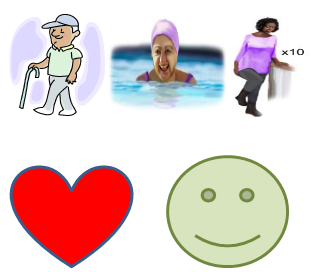  **** | We want to support **people** to be **more active** after a **stroke,** once the **NHS rehabilitation** has **stopped**  This will **help** keep **stroke** survivors **healthy**  We will **train** **therapists** to provide **therapy in a new way** to help **stroke survivors** be **more active** |
| 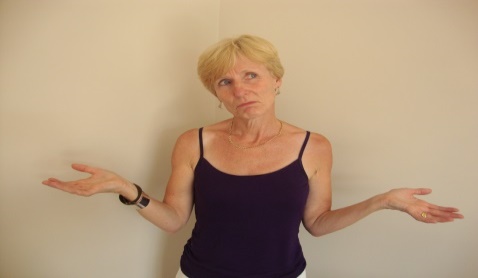  **** | **We don’t know** if this will **work**  This is what **the research** will **try to find out** |
| 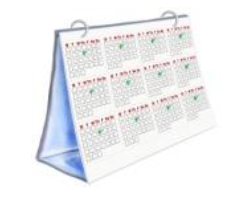 | The **whole research** will last for **18 months** |
| **** | You will have at least 2 **appointments with a therapist**  The appointments will **match with the dates** of your **normal therapy** |

**Why have you been asked to take part?**

| 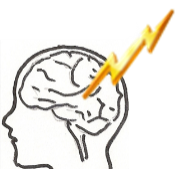 | You have **had a stroke** |
| --- | --- |
| 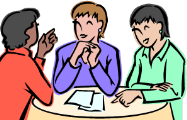   | You are having **rehabilitation** |
| **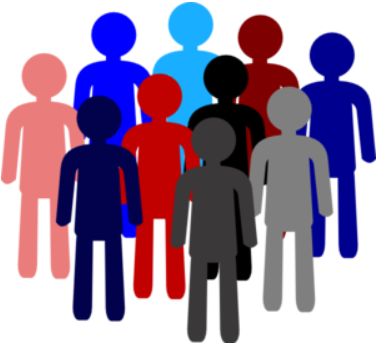** | We are asking lots of **other people** too |

**Do I have to take part?**

| 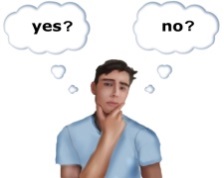  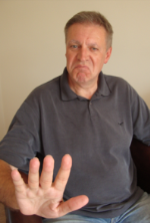 | **You can decide**  **You do not have to take part** in the research if you do not want to |
| --- | --- |
| 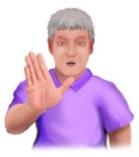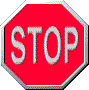  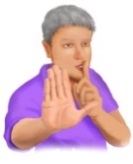  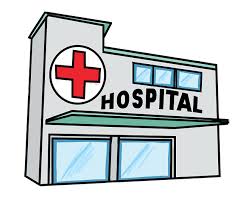 | If you change your mind, **you can stop** at any time  You **don't** have to **give a reason**  **If you stop** this will **not affect** your **normal care and treatment** |
| 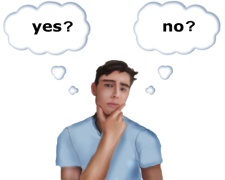  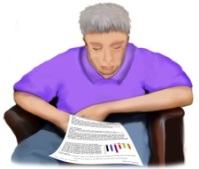  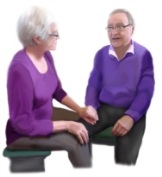 | You don't have to decide now, you can **think about it**  You can **take your time**  You can  **read** the information **again**  You can **talk to your family** to **help you decide** |

**What happens in the research?**

| We will tell your **doctor** that you are in this study.      A **therapist** will visit you in your home  ****  The **therapist** will be member of the **stroke rehabilitation team**    The **therapist** will ask you to complete **questionnaires**   - About how **tired** you generally feel **(fatigue)**     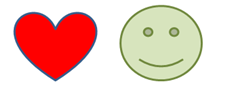   - About your **well-being**      - Your **mobility**   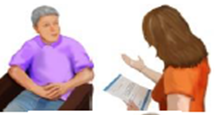  The **therapist** will ask you **questions** about:  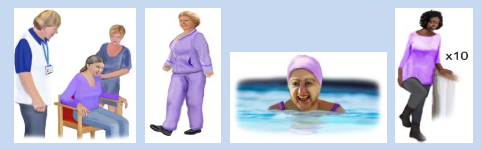   - **Types** of activities you may like to do      - Why you **want** to do the **activities**     The therapist will help you **plan goals** to increase your **activity**      **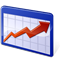**  **Teach** ways to **record** the **activities** you **do.**  This is called **monitoring your progress**    Give you **information** about **services** in  **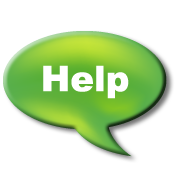**  your **local area** to help you be **more active** |
| --- |
| The **therapist** will make **sound recordings** of what is **said to you** about doing more **activity**    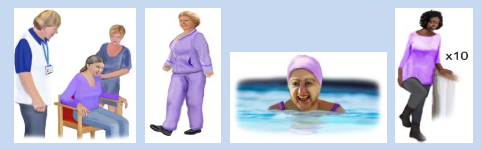    ****  This will **help** us to **train** the **therapist**    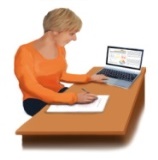  **Only the researchers** will **hear the sound**  **recordings**      The **sound recordings** will be kept **safe**    We may also **ask you** take part in an **interview** with a **researcher**  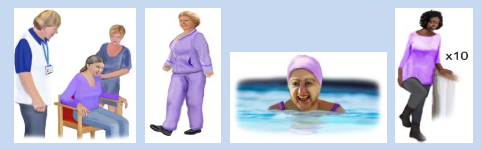  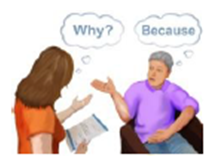  The **researcher** will ask you about your **experiences** and **opinions** about the **research**  [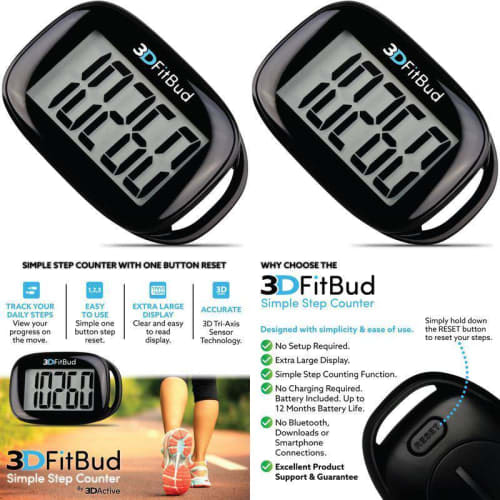](https://www.google.com/url?sa=i&rct=j&q=&esrc=s&source=images&cd=&ved=2ahUKEwjs8sr44ZbdAhVDyYUKHfAaAIsQjRx6BAgBEAU&url=https://www.ebay.co.uk/itm/3D-Fitbud-Simple-Step-Counter-Walking-3D-Pedometer-With-Lanyard-A420S-Black-/282885393060&psig=AOvVaw2xjLIPUMXIUPViNmpSBFcl&ust=1535786825897195)  You can **keep** any **tools** we give you to help you be more active **e.g. a step counter** |

**Will I get paid?**

| **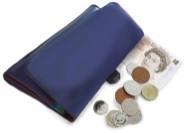X** | You will **not get paid** for taking part in the research |
| --- | --- |

**What might be good about taking part?**

| 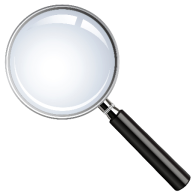  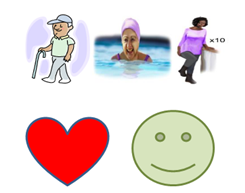  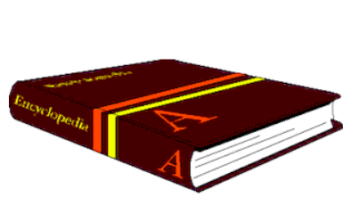 | You will **help**  people in the **future**  You will help us to **learn**  You could become **more active** and *healthier* (although **we don’t know** this for sure)  This may **provide better treatment for strokes** in the **future** |
| --- | --- |

**What might be difficult about taking part?**

|  | When **asked** about **activities** you will need to discuss your **difficulties**  Some people may find this **upsetting** |
| --- | --- |
| 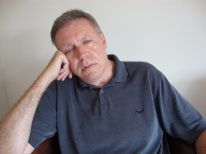 | You may find it **tiring** |
| 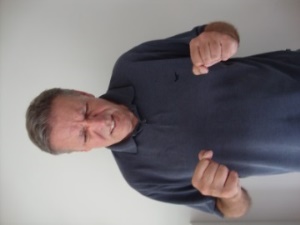 | You may be **frustrated** if there is **no progress**  You may be **frustrated** if **progress is slow** |

**What if I don’t want to continue with the research?**

| 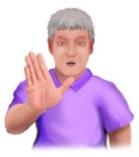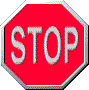 | If you **do** **take part** in the research you **can stop** at any time. | |
| --- | --- | --- |
| **If you stop** being in **the research….** | | |
| 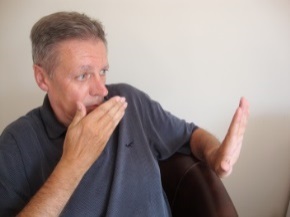 | | You **don’t** have to **give a reason**. |
| Normal therapy   | | Your **normal therapy** will **not** be **affected** |
|  | | Any **information already collected** about you will **still be used** **unless you ask** for this **not to happen** |

**What if something goes wrong?**

| If there is a problem **contact**  your local stroke team on  **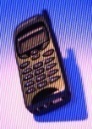** xxxx 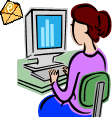 xxxxx@ .com |
| --- |

| or Dr **Sarah Moore** who is leading this **research**  Stroke Research Group  Institute of Neuroscience  Newcastle University  3-4 Claremont Terrace  Newcastle upon Tyne  NE2 4AE | 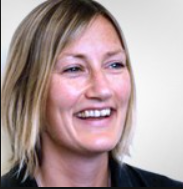  **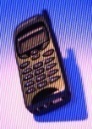** **0191 208 3837**  [**s.a.moore@ncl.ac.uk**](mailto:s.a.moore@ncl.ac.uk) |
| --- | --- |
| If you remain **unhappy** and wish to **complain** about the study you can contact your local **Patient Advisory Liaison Service:**  *[local contact details for PALS to be added to local version PIS]* | \|  \| \| --- \| \|  \| |

**Who will see the information about me?**

| 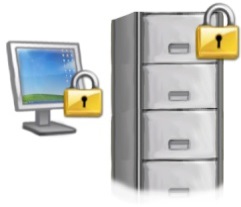 | We will keep the **information** about you **safe**  **Only**  the  **PARAS researchers** will see the information about you |
| --- | --- |

| **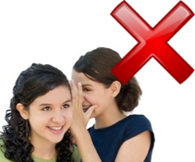** | The information will **not be shared** with people outside the study |
| --- | --- |

**What will happen after the research?**

| 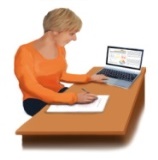 | The **researchers** will **look at the results** |
| --- | --- |

|  | They will **share the results** with the **people who** **paid** for the research – The National Institute for Health Research |
| --- | --- |
|  | They will **share** the **results** with **other researchers** |
| 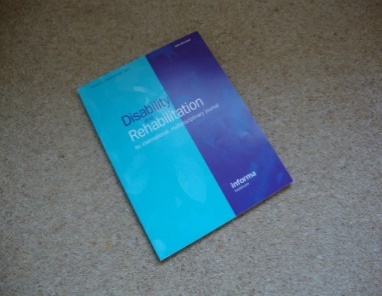 | They will **write** about the results in **academic magazines** (journals) |

| Name…..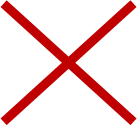 | The results will **not use your name** |
| --- | --- |

**Who is doing the research?**

| ***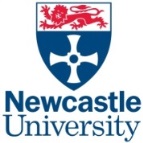*** | The research is run from **Newcastle University** and **Northumbria Healthcare** **NHS Foundation Trust** |
| --- | --- |
| 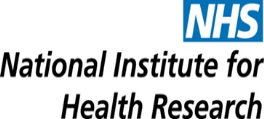 | The **National Institute for Health Research** is **paying**  for this research |

**Who has reviewed the research?**

| 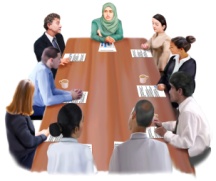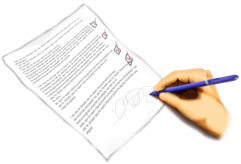 | A committee **decides if** **research can happen** This is the **ethics committee**  This **research** has been reviewed by  **North East – Tyne & Wear South Research Ethics Committee** |
| --- | --- |

**What next?**

|  | We can **answer questions** you might have **about the research** |
| --- | --- |
| 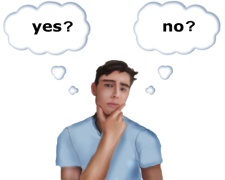 | Think about whether you **want to take part** in the research |
| **** | If you decide to take part you will need to **sign a consent form** |

**Appendix E Workshop findings – stroke survivors**

**PARAS mapping workshop**

**Workshop 1**

**General feedback**

1. **Workbook**

- **Benefits**
- Content well organised strongly agree 67%, agree 25%, undecided 8%
- Content easy to follow strongly agree 83%, agree 17%
- Design appropriate strongly agree 75%, agree 25%
- Categories cover all areas and are clear and simple
- ‘Illness’ descriptors should be broken down.
- Separate cardiovascular disease and diabetes
- Change some of icons: cancer ribbon should be pink; long life should be a battery and a plus; scales disagreement between if it should be old or new scales; stress and anxiety mouth going down to mouth going up into smile; getting out of the house and meeting people: people sat around a table; reduces dependence someone walking away from someone.
- **Outcomes**
- Content well organised strongly agree 100%
- Content easy to follow strongly agree 100%
- All quotes relevant.
- Swap two quotes for engaging in meaningful activities around
- Change wording ‘to get back to pre-stroke self’ to ‘finding new ways of doing activities I used to enjoy’…look at quotes.
- **Planned activities**
- Content well organised strongly agree 58%, agree 33%, undecided 9%
- Content easy to follow strongly agree 58%, agree 42%
- Design appropriate strongly agree 64 %, agree 36%
- No activities missing 64% strongly agree, 36%
- Change chair based exercises to person in a chair doing exercises
- Change home based exercise programme to someone lifting a can of beans
- **Goal setting**
- Content well organised strongly agree 70%, agree 20%, undecided 10 %
- Content easy to follow strongly agree 70%, agree 20% undecided 10%
- Design appropriate strongly agree 64 %, agree 36%
- Usefulness of goal sheets strongly agree 80%, agree 10% undecided 10%
- Helpful and clear
- Liked goal should be just out of reach but not out of sight
- Goal sheet clear and appropriate
- Measuring success easy to follow
- ‘Excellent section. Examples will give people ideas of the goals they can set for themselves’.
- **Barrier identification**
- Content well organised 56% strongly agree, 33% agree, 11% undecided
- Content easy to follow 44% strongly agree, 44% agree 12% undecided
- Design is appropriate 44% strongly agree, 34% agree, 11% undecided, 11% disagree

**Reviewing progress and future plans**

- Content well organised strongly agree 56%, 33% agree, 11% disagree
- Content easy to follow strongly agree 56%, agree 22%, undecided 22%
- Design is appropriate strongly agree 45%, agree 33%, undecided 22%
- Success of example strongly agree 100%
- Suggested first review session is face to face, perhaps telephone half way between to check on progress.
- Everyone has to have at least one review (could be telephone or face to face)
- Option for more review sessions if appropriate.
- Review checklist clear
- **General instructions**
- Content well organised 84% strongly agree, 8% agree, 8% agree
- Content easy to follow 90% strongly agree, 10% agree
- Design is appropriate 90% strongly agree 10% undecided
- Clear. Would be helpful to have in workbook (as important and some people won’t have internet) but also as a video on website.
- **Goal examples**
- Clear and useful. Three right number but might be useful to have more examples on website relating to all outcomes.

1. **PA diaries**

- Likely to use strongly agree 33%, agree 33%, undecided 25% , disagree 9%
- ‘Very useful for a carer maybe more than a stroke survivor can complete, useful to show whether goals have been met or not’.

1. **Apps**

- Phone capacity 20% strongly agree, 20% agree, 60% undecided
- Regular app download 20% strongly agree, 20% undecided, 60% disagree
- Suitability for stroke survivors 33% strongly agree, 67% strongly disagree
- Use of app 33% strongly agree, 67% strongly disagree

1. **Pedometers**

- CSX favourite pedometer and was voted most easy to use/likely to use
- Omron writing too small, buttons hard to push
- Mountain warehouse easier to set up than some others as not many steps writing clearer and buttons easier to push.
- 3D trisport writing clear and buttons easy to press not as flimsy as others ones. Good to have an option to wear around neck or clip to belt.
- Pingko display very clear a good size for wearing set and reset mode difficult to see when first using it
- Might be useful to have two pedometers to offer one that has very basic functionality and one that has calories etc.
- When accounting for stroke survivor preference, cost and field based testing CSX pedometer rated most favourably

Results of field-based testing of pedometers with simulated gait speeds

| Pedometer | Cost (£) | Position | Usual gait speed | Slow gait speed (<0.5 m/s) |
| --- | --- | --- | --- | --- |
|  |  |  | Actual step count/pedometer measured step count | Actual step count/pedometer measured step count |
| 3D Trifit | 9.99 | Right hip | 22 vs. 23 | 22 vs. 0 |
| Pingko | 6.99 | Right hip | 22 vs. 22 | 22 vs. 0 |
| CSX 301S 3D simple pedometer | 11.99 | Right hip | 22 vs. 25 | 22 vs. 0 |
| CSX301S 3D simple pedometer | 11.99 | neck | 22 vs. 25 | 22 vs. 25 |
| Digiwalker (used in Pace up study(Harris, Kerry et al. 2017)) | 21.50 | Right hip | 22 vs. 30 | 22 vs. 26 |
| Mountain warehouse | 3.49 | Right hip | 22 vs. 25 | 22 vs. 25 |
| Omron HJ-321-E (Carroll, Greig et al. 2012) | 24.49 | Right hip | 22 vs. 22 | 22 vs 0 |
| 3D trisport | 16.99 | Right hip | 22 vs. 25 | 22 vs. 25 |
| 3D tripsort | 16.99 | neck | 22 vs. 23 | 22 vs. 26 |
| 330 step pedometer sportline (Sullivan, Espe et al. 2014) | 13.23 | Right hip | 21 vs. 22 | 21 vs. 14 |

1. **Overall views of intervention**

- Content easy to follow 75% strongly agree, 12.5 % agree, 12.5% undecided
- Design is appropriate 67% strongly agree, 22% agree, undecided 11%
- Right amount of information 75% strongly agree, 12.5% agree, 12.5% undecided

Carroll, S. L., C. A. Greig, S. J. Lewis, M. E. McMurdo, F. F. Sniehotta, M. Johnston, D. W. Johnston, J. Scopes and G. E. Mead (2012). "The Use of Pedometers in Stroke Survivors: Are They Feasible and How Well Do They Detect Steps?" Archives of Physical Medicine and Rehabilitation **93**(3): 466-470.

Harris, T., S. M. Kerry, E. S. Limb, C. R. Victor, S. Iliffe, M. Ussher, P. H. Whincup, U. Ekelund, J. Fox-Rushby, C. Furness, N. Anokye, J. Ibison, S. DeWilde, L. David, E. Howard, R. Dale, J. Smith and D. G. Cook (2017). "Effect of a Primary Care Walking Intervention with and without Nurse Support on Physical Activity Levels in 45- to 75-Year-Olds: The Pedometer And Consultation Evaluation (PACE-UP) Cluster Randomised Clinical Trial." PLOS Medicine **14**(1): e1002210.

Sullivan, J. E., L. E. Espe, A. M. Kelly, L. E. Veilbig and M. J. Kwasny (2014). "Feasibility and Outcomes of a Community-Based, Pedometer-Monitored Walking Program in Chronic Stroke: A Pilot Study." Topics in Stroke Rehabilitation **21**(2): 101-110.
